# SNP‐based genotyping and whole‐genome sequencing reveal previously unknown genetic diversity in *Xanthomonas vasicola* pv. *musacearum*, causal agent of banana xanthomonas wilt, in its presumed Ethiopian origin

**DOI:** 10.1111/ppa.13308

**Published:** 2020-11-27

**Authors:** Gloria V. Nakato, David J. Studholme, Guy Blomme, Murray Grant, Teresa A. Coutinho, Evans M. Were, Emmanuel Wicker, George Mahuku

**Affiliations:** ^1^ Plant Pathology International Institute of Tropical Agriculture Kampala Uganda; ^2^ Department of Microbiology and Plant Pathology Centre for Microbial Ecology and Genomics (CMEG), Forestry and Agricultural Biotechnology Institute (FABI) University of Pretoria Pretoria South Africa; ^3^ Biosciences University of Exeter Exeter UK; ^4^ Bioversity International, c/o ILRI Addis Ababa Ethiopia; ^5^ Life Sciences University of Warwick Coventry UK; ^6^ CIRAD UMR Interactions Plantes‐Microorganismes‐Environnement (IPME) Montpellier cedex 5 France; ^7^ IPME University of Montpellier CIRAD Montpellier cedex 5 France; ^8^ International Institute of Tropical Agriculture (IITA) Dar es Salaam Tanzania

**Keywords:** *Ensete ventricosum*, genomics, *Musa acuminata*, population, RFLP, xanthomonas wilt

## Abstract

For decades, *Xanthomonas vasicola* pv. *musacearum* (Xvm) has been an economically important bacterial pathogen on enset in Ethiopia. Since 2001, Xvm has also been responsible for significant losses to banana crops in several East and Central African countries, with devastating consequences for smallholder farmers. Understanding the genetic diversity within Xvm populations is essential for the smart design of transnationally reasoned, durable, and effective management practices. Previous studies have revealed limited genetic diversity in Xvm, with East African isolates from banana each falling into one of two closely related clades previously designated as sublineages SL 1 and SL 2, the former of which had also been detected on banana and enset in Ethiopia. Given the presumed origin of Xvm in Ethiopia, we hypothesized that both clades might be found in that country, along with additional genotypes not seen in Central and East African bananas. Genotyping of 97 isolates and whole‐genome sequencing of 15 isolates revealed not only the presence of SL 2 in Ethiopia, but additional diversity beyond SL 1 and SL 2 in four new clades. Moreover, SL 2 was detected in the Democratic Republic of Congo, where previously SL 1 was the only clade reported. These results demonstrate a greater range of genetic diversity among Xvm isolates than previously reported, especially in Ethiopia, and further support the hypothesis that the East/Central Africa xanthomonas wilt epidemic has been caused by a restricted set of genotypes drawn from a highly diverse pathogen pool in Ethiopia.

## Graphical abstract

East and Central Africa xanthomonas wilt epidemic has been caused by a restricted set of genotypes drawn from a highly diverse pathogen pool in Ethiopia.

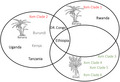

## 1 INTRODUCTION

Within the γ‐proteobacteria, the genus *Xanthomonas* consists of more than 20 species and over 100 different pathovars causing several economically important diseases of plants (Vauterin et al., 1995). Among these pathogens is *Xanthomonas vasicola* pv. *musacearum* (Xvm), the causative agent of xanthomonas wilt of banana and enset (Nakato et al., 2017; Studholme et al., 2020). Xvm is a vascular pathogen (Yirgou & Bradbury, 1968, 1974), principally transmitted by insects (Tinzaara et al., 2007), contaminated tools (Addis et al., 2010), and infected planting material (Biruma et al., 2007). Disease symptoms on banana and enset are characterized by rapid yellowing and wilting of leaves, bacterial ooze in the pseudostem and leaf sheaths/petioles/midribs, and premature ripening and internal discolouration of the fruit (Blomme et al., 2017; Eden‐Green, 2004).

Xvm was initially described in Ethiopia on *Ensete ventricosum* (Castellani, 1939; Yirgou & Bradbury, 1968) and subsequently on banana (Yirgou & Bradbury, 1974) as *Xanthomonas musacearum*. The pathogen's taxonomic position was recently revised, placing it within the species *X*. *vasicola* (Studholme et al., 2020). The pathogen has been reported since 2001 on banana in banana‐producing regions of Eastern and Central Africa (ECA), first in Uganda (Tushemereirwe et al., 2004), then the Democratic Republic of Congo (D. R. Congo; Ndungo et al., 2006), Tanzania (Carter et al., 2010), Rwanda (Reeder et al., 2007), Burundi (Carter et al., 2010), and Kenya (Carter et al., 2010). Despite these country‐level reports, there is no detailed geographical/temporal spread information available for most countries in the ECA region. Genetic relationships between bacterial isolates from different times and places can be used to infer chains of pathogen transmission. However, previous studies have revealed very limited genetic diversity in Xvm (Aritua et al., 2007; Odipio et al., 2009).

Whole‐genome sequencing revealed two closely related clades, or sublineages (SL), that appeared to be geographically separated (Wasukira et al., 2012): isolates from Ethiopia, D. R. Congo, and Rwanda fell into SL 1, whereas isolates from Burundi, Kenya, Tanzania, and Uganda belonged to SL 2. Because Xvm was reported in Ethiopia decades before it was detected in Uganda and thereafter other ECA countries, it was widely assumed that the East African epidemic originated from Ethiopia. However, to date only SL 1, and not SL 2, has been reported in Ethiopia. Furthermore, if Ethiopia were the centre of origin, then it should harbour an even greater genetic diversity of Xvm in addition to the two previously known clades.

To test this hypothesis, we genotyped a large collection of isolates from a range of geographical locations in Ethiopia and Central and East Africa, using eight single‐nucleotide polymorphisms (SNPs) previously reported to distinguish SL 1 from SL 2 (Wasukira et al., 2012). The genetic diversity of Xvm populations in East and Central Africa has been explored previously using the multilocus variable number of tandem repeat analysis (MLVA; Nakato et al., 2019). In that study, the discriminatory powers and congruence of MLVA and SNP typing techniques were assessed and compared. Results showed that the MLVA haplotypes corresponded to the SNP haplotypes and were consistent with the SNP‐based SLs (Nakato et al., 2019). It was determined that both MLVA and SNPs can be used together within a hierarchical typing procedure. However, the phylogenetic relationship of the SNP haplotypes remains to be determined and clarified by additional genomic sequence analysis. We therefore sequenced the genomes of 15 Xvm isolates representing the full range of haplotypes, and carried out a phylogenetic reconstruction from genome‐wide data.

## 2 MATERIALS AND METHODS

### 2.1 Collection of samples from Xvm‐infected plants

Most of the Xvm isolates from banana (*Musa acuminata* × *M*. *balbisiana*) and enset (*E. ventricosum*) were collected between December 2014 and February 2015 from Ethiopia, D. R. Congo, Rwanda, Tanzania, and Uganda; some Ethiopian Xvm isolates (*n* = 12) were also collected from enset during an earlier survey in 2004. Ninety‐seven isolates were collected from D. R. Congo (*n* = 7; all isolated from banana), Ethiopia (*n* = 20; one isolated from maize, 19 isolated from enset), Rwanda (*n* = 4; all isolated from banana), Tanzania (*n* = 16; all isolated from banana), and Uganda (*n* = 50; all isolated from banana). Thus, 77 isolates were isolated from banana, 19 from enset, and 1 from maize. Within each country, samples were collected from different locations that were approximately 5 km apart. Samples were collected from corms and pseudostems of diseased banana plants, and from pseudostems and leaf petioles of diseased enset. Sampling tools were sterilized between samples to avoid cross‐contamination. For each sample collected, we recorded the GPS coordinates, altitude, and name of the cultivar from which the sample was taken.

### 2.2 Isolation of Xvm

Three grams of the sample was homogenized in 3 ml of sterile distilled water containing Tween 80 (0.02% vol/vol) using a sterile mortar and pestle and 1 ml of the homogenate was serially diluted with sterile distilled water. A 20 µl aliquot from the 10^−2^ dilution was spread on three 9 cm Petri plates containing modified YPGA (containing per L of distilled water: yeast extract 5 g, peptone 5 g, glucose 10 g, agar 15 g, 5‐fluorouracil 15 mg, cephalexin 45 mg; Mwangi et al., 2007). Petri plates were sealed with Parafilm and incubated at 28 °C for 4 days. Colonies with Xvm‐like characteristics were transferred to Petri plates containing YDCA (containing per L of distilled water: yeast extract 2.5 g, dextrose 5 g, calcium carbonate 15 g, agar 14 g; Mwangi et al., 2007).

All purified isolates were stored at −80 °C in glycerol stocks (20% glycerol + 80% YPG broth). The final collection consisted of 97 isolates.

### 2.3 Bacterial DNA extraction

Total DNA was extracted from Xvm‐like colonies using a small‐scale protocol described by Mahuku (2004). Briefly, a loopful of 3‐day‐old Xvm cells were harvested and washed twice in 500 μl of 1 M NaCl in Eppendorf tubes to reduce and separate the Xvm cells from the polysaccharide xanthan gum. The bacterial cells were washed twice with sterile distilled water to reduce salt concentration. The bacterial cell pellets were suspended in 500 μl of prewarmed (55 °C) TES extraction buffer (0.2 M Tris‐HCl, pH 8, 10 mM EDTA, 0.5 M NaCl, 1% SDS) containing proteinase K (50 μg/ml), vortexed for 30 s, and incubated at 65 °C for 15 min. One‐half volume (250 µl) of 7.5 M ammonium acetate was added, gently mixed, and the samples left to stand for 10 min at room temperature. Tubes were centrifuged at 11,830 × *g* for 15 min and 500 μl of the supernatant transferred to a fresh tube. The DNA was precipitated by adding an equal volume (500 μl) of ice‐cold isopropanol, gently mixing and incubating at −20 °C overnight. Tubes were centrifuged at 15,750 × *g* and 4 °C for 10 min and the DNA pellet was washed with 800 μl of cold 70% ethanol. The DNA pellet was air dried by inverting tubes on clean paper towels for 30 min at room temperature. The DNA pellet was resuspended in 100 μl of nuclease‐free water. Integrity of DNA was determined using the NanoDrop 2000C spectrophotometer (Thermo Fisher Scientific Inc.), adjusted to 50 ng/μl, and stored at −20 °C until use.

### 2.4 PCR‐RFLP genotyping

Among the 86 SNPs discriminating SL 1 from SL 2 (Wasukira et al., 2012), eight were used to classify Xvm isolates into sublineages. Restriction fragment length polymorphism (RFLP) assays for four SNP markers (Was1 to Was4) were published previously (Wasukira et al., 2012), while four others were developed in the present study. These SNPs fell within genes encoding a hypothetical protein, urocanate hydratase, and within intergenic regions. PCR amplifications of target DNA were conducted in 20 μl reaction volumes containing 50 ng genomic DNA, 1 U *Taq* DNA polymerase (Bioneer Corporation), 6 pmol of each of the primers (Table 1), 0.2 mM dNTPs, 2 mM MgCl_2_, and 1× reaction buffer (Promega). PCR amplifications were performed on a Techne thermocycler with initial denaturation for 5 min at 95 °C; 35 cycles of 30 s denaturation at 95 °C, 30 s annealing (see Table 1 for Tm of primers), 30 s extension at 72 °C; and a final extension for 10 min at 72 °C. Each set of primers specified a target ranging from 237 to 590 bp. The exception was the EW4F/EWR primer pair, which targeted a 1,000‐bp sequence that contained the 500‐bp target of the WAS4F/WAS4R primers. Primers EW4F/EW4R were designed on these extended loci using Geneious (Biomatters) (Kearse et al., 2012). The designed primers were tested in silico for primer efficiency and ability to amplify using Geneious v. 9.1 software (Kearse et al., 2012), and then purchased from BIONEER Inc. or Eurogentec (Angers, France).

**Table 1:** The eight single‐nucleotide polymorphisms used for genotyping *Xanthomonas vasicola* pv. *musacearum* isolates

SNP	Restriction site	Primer	Primer sequence (5′–3′)	Target sequence RefSeq accession number and coordinates	Expected amplicon size (bp)	Annealing temperature (°C)
KB372850.1:82,543	*Alu*I (AG↓CT)	WAS1F	GAGCTCCTGCGCCGATGC	KB372850.1:82,293–82,791	498	53
		WAS1R	GTGAGCGTAAAGGCGGCTATTCTA			
KB372851.1:40,710	*Fok*I (GGATG(N)_9_↓)	WAS2F	CGGCGTGGTTTTGCCTTTGC	KB372851.1:40,460–40,926	484	53
		WAS2R	CGTACGGCCTGGCGGTGAT			
KB372868.1:106,342	*Alu*I (AG↓CT)	WAS3F	TCACCTGTTCGATGCGGCC	KB372868.1:106,092–106,566	488	67
		WAS3R	GCTACTGGCTGTCGCGGC			
KB372852.1:30,841	*Nde*I (CA↓TATG)	WAS4F	ATGTTTGCCGATACCTGGATGC	KB372852.1:30,604–31,091	487	63
		WAS4R	GCATGCTTGCCGGTTTCGACGA			
		EW4F	CATGGCGATCAGACCCACCGTG	KB372852.1:30,436–31,263	828	62
		EW4R	AGGTACTCGAAATCATCCTGCGGG			
KB372860.1:47,818	*Hpy*188III	VN2F	GCGCTTCGATGGGTTGCACA	KB372860.1:47,522–48,111	590	60
	(TC↓CCGA)	VN2R	ACAAACCCTTGCGCACGACC			
KB372875.1:31,413	*Asc*I	VN5F	AAAACCTGCAACGCACCGCA	KB372875.1:31,129–31,692	564	60
	(GG↓CGCGCC)	VN5R	AGCACCGACTTCTCCCGCAT			
KB372864.1:11,305	*Mlu*I	VN11F	TGCGCGTCGGCAGTGTGATA	KB372864.1:11,154–11,390	237	60
	(A↓CGCGT)	VN11R	GTTCAAGCGCAACGGCACCT			
KB372873.1:18,761	*Afl*III	VN12F	CATCCAGGTGCGGATTGTTC	KB372873.1:18,410–18,883	474	60
	(A↓CACGT)	VN12R	TGATTCCTACCGCAGTCGAG			

Note.

The WAS primers were described previously (Wasukira et al., 2012). The EW4 and VN primers were developed in this study.

Amplified PCR products were separated on 2% (wt/vol) agarose gels in 1× TAE buffer at 150 V for 40 min. The gels were stained with ethidium bromide (0.5 μg/ml) and gel images captured using the GBOX gel documentation system (Syngene). Subsequently, 5 µl aliquots of PCR products were digested with the restriction endonucleases (New England Biolabs) detailed in Table 1. Restricted DNA was separated by electrophoresis in 1.5% agarose gels and visualized as previously described.

### 2.5 DNA sequencing and phylogenomic analysis

Genomic DNA was sequenced using the Illumina MiSeq according to the manufacturer's instructions. Sequence reads were filtered and trimmed using the TrimGalore wrapper for CutAdapt (Martin, 2011) before analysis using REALPHY (Bertels et al., 2014) with RaxML (Stamatakis, 2014) as the tree‐construction method. Genome sequences were assembled using SPAdes v. 3.11.1 (Bankevich et al., 2012) and annotated with the Prokaryotic Genomes Annotation Pipeline (PGAAP) at the NCBI (Tatusova et al., 2016; Haft et al., 2018). Genome sequence data are available via BioProject accession number PRJNA454153. Summary statistics and a full list of GenBank and Sequence Read Archive accession numbers are listed in Table S1. We identified open reading frames encoding Type III effectors by performing TBLASTN searches against each genome assembly using the effector amino acid sequences from the *Xanthomonas* Resource website (http://xanthomonas.org/t3e.html). We considered an effector gene to be present if the TBLASTN alignment covered at least 85% of the query length with an amino acid identity of at least 70%.

### 2.6 Pan‐genome analysis

The pan‐genome was calculated using Roary v. 3.13.0 (Page et al., 2015) after annotating the genome assemblies with Prokka v. 1.14.5 (Seemann, 2014).

### 2.7 Definition of haplotypes

Table 2 summarizes the nomenclature of the haplotypes across eight SNP loci and the corresponding clades (sublineages).

**Table 2:** Naming of the haplotypes observed in this study and their correspondence to Wasukira's sublineages

Haplotype	WAS1	WAS2	WAS3	WAS4/EW4	VN2	VN5	VN11	VN12	Wasukira's sublineage	Clade (this study)	No. of isolates sharing WAS haplotypes	No. of isolates sharing WAS + VN haplotypes
1	Nc	Nc	C	Nc	Nc	Nc	Nc	C	SL 1	1	11	8
2	C	C	C	C	C	C	C	Nc	SL 2	2	67	8
3	C	C	C	Nc	NT	NT	NT	NT	—	6	14	0
3a	C	C	C	Nc	Nc	C	2c^a^	Nc	—	2	0	2
3b	C	C	C	Nc	Nc	Nc	2c^a^	Nc	—	3, 5	0	5
4	C	Nc	C	Nc	Nc	Nc	2c^a^	Nc	—	4	5	4

Note.

The entire collection (*n* = 97) was genotyped using the WAS1–WAS4 markers, and a subset of 27 isolates was also genotyped using the four new VN markers. For each PCR‐RFLP assay listed in Table 1, the result was either cleavage by the restriction enzyme ‘C’, or no cleavage ‘Nc’. Haplotypes 3a and 3b share the same WAS1–WAS4 pattern, and strains genotyped with WAS1–WAS4 only and showing this pattern were thus assigned to Haplotype 3. Haplotypes 1 and 2 correspond to the previously described SL 1 and SL 2 sublineages (Wasukira et al., 2012), while haplotypes 3 and 4 have not previously been observed and are inconsistent with both SL 1 and SL 2. Haplotypes were determined based on the SNP using a PCR‐RFLP assay and clades were determined based on genome based‐phylogenetic analysis. NT, not tested.

^a^ Restriction was achieved at two restriction sites, yielding three bands.

### 2.8 Haplotype network analysis

Haplotypes across 1,655 SNPs were inferred from the resulting alignment using a custom script (available at https://github.com/davidjstudholme/SNPsFromPileups) from alignments of genomic sequence reads against the NCPPB 4379 reference genome sequence (GenBank CP034655.1), generated using BWA‐mem (Li & Durbin, 2009). This resulted in a Nexus‐formatted output file. This Nexus (Maddison et al., 1997) file served as input into Popart (Leigh & Bryant, 2015) to generate the median‐joining network (Bandelt et al., 1999).

## 3 RESULTS

### 3.1 Collection and identification of isolates

All isolates were identified as Xvm by PCR amplification of five Xvm‐specific coding sequences (Nakato et al., 2018) and preserved as glycerol stocks at −80 °C. The entire collection (*n* = 97) was genotyped using the four Wasukira's WAS1–WAS4 markers, and a subset of 27 isolates was also genotyped using the four new RFLP markers (VN2, VN5, VN11, VN12) (Table 2).

### 3.2 Genotyping of Xvm isolates by PCR‐RFLP

Most of the isolates yielded unambiguous PCR‐RFLP results with all eight primer pairs. The exceptions were two isolates from D. R. Congo that failed to amplify with the WAS4 primers (D13L and D24L; Table 3). In order to resolve the genotype at the WAS4 SNP, we designed a new primer pair (EW4F/R) that successfully amplified a product of the expected size for these isolates.

**Table 3:** Details of geographical location, year, host of isolation, and haplotype for the 27 *Xanthomonas vasicola* pv. *musacearum* isolates that were characterized using the eight single‐nucleotide polymorphism (SNP) markers

Country	Village/ward	Isolate	Altitude (m a.s.l.)	Latitude	Longitude	Host	Year of isolation	Haplotype
D. R. Congo	Kabamba	D13L^a^	1,553	2.197	28.880	Banana	2015	1
D. R. Congo	Mbinga sud	D24L^a^	1,589	2.072	28.898	Banana	2015	1
D. R. Congo	Irambi	D34L	1,519	2.187	28.856	Banana	2015	1
D. R. Congo	Irambi	D35L	1,519	2.187	28.856	Banana	2015	1
D. R. Congo	Walungu	D46L	1,754	2.593	28.722	Banana	2015	2
Ethiopia	Sodo Zuria	BCC210	1,670	6.833	37.749	Enset	2004	3a
Ethiopia	Awassa	BCC246	1,680	7.050	38.495	Enset	2004	3b
Ethiopia	Sodo Zuria	BCC247	2,100	6.833	37.749	Enset	2004	4
Ethiopia	Sodo Zuria	BCC248	2,100	6.833	37.749	Enset	2004	4
Ethiopia	Lemu	BCC250	2,670	7.600	39.217	Enset	2004	4
Ethiopia	Loma Bosa	BCC265	2,060	6.916	37.333	Maize	2004	4
Ethiopia	Loma Bosa	BCC267	2,060	6.916	37.333	Enset	2004	1
Ethiopia	Kochere	BCC274	2,160	6.000	38.249	Enset	2004	3a
Ethiopia	Hagere Selam	BCC278	2,660	6.488	38.521	Enset	2004	3b
Ethiopia	Hagere Selam	BCC280	2,550	6.488	38.521	Enset	2004	3b
Ethiopia	Hagere Selam	BCC281	2,550	6.488	38.521	Enset	2004	3b
Ethiopia	Amaro	BCC282	1,800	5.827	37.723	Enset	2004	3b
Rwanda	Mutete	R1P	1,680	−1.674	30.092	Banana	2015	1
Rwanda	Buhoro	R2L	1,801	−2.188	29.775	Banana	2015	1
Rwanda	Ruharambuga	R5P	1,590	−2.448	29.041	Banana	2015	1
Tanzania	Itongo	T31C	1,212	−1.503	31.581	Banana	2015	2
Tanzania	Itongo	T33C	1,212	−1.503	31.581	Banana	2015	2
Tanzania	Nyakabanga	T40C	1,197	−1.573	31.542	Banana	2015	2
Tanzania	Kyaitoke	T41C	1,221	−1.549	31.453	Banana	2015	2
Uganda	Rugendabara	AS50C	1,119	0.352	30.194	Banana	2014	2
Uganda	Kasaala	AS83C	1,100	0.891	32.480	Banana	2014	2
Uganda	Kasaala	SY84P	1,100	0.891	32.480	Banana	2014	2

^a^ Isolates from D. R. Congo that failed to amplify with the WAS4 primers.

Of the 2^8^ (256) haplotypes theoretically possible over four biallelic SNPs, four were observed in the present study. These haplotypes are summarized in Table 2 and haplotypes of each isolate are listed in Table 3. Haplotypes 1 and 2 were identical to those described for sublineages SL 1 and SL 2, respectively (Wasukira et al., 2012). However, Haplotypes 3 and 4 did not match the haplotypes of any of the previously sequenced Xvm genomes (Studholme et al., 2010; Wasukira et al., 2012; Table 2). For Haplotype 3, restriction was observed in all PCR products except for WAS4. The VN primers further discriminated two patterns within the Haplotype 3 isolates, the locus VN5 being cut by the restriction enzyme *Asc*I or not, separating Haplotype 3a and Haplotype 3b, respectively. In addition, results support that Haplotype 3, as defined by WAS markers, may be subdivided into at least two haplotypes. Haplotype 3 isolates that were not tested by VN markers could show the 3a profile, the 3b profile, or another profile.

**Figure 1:**
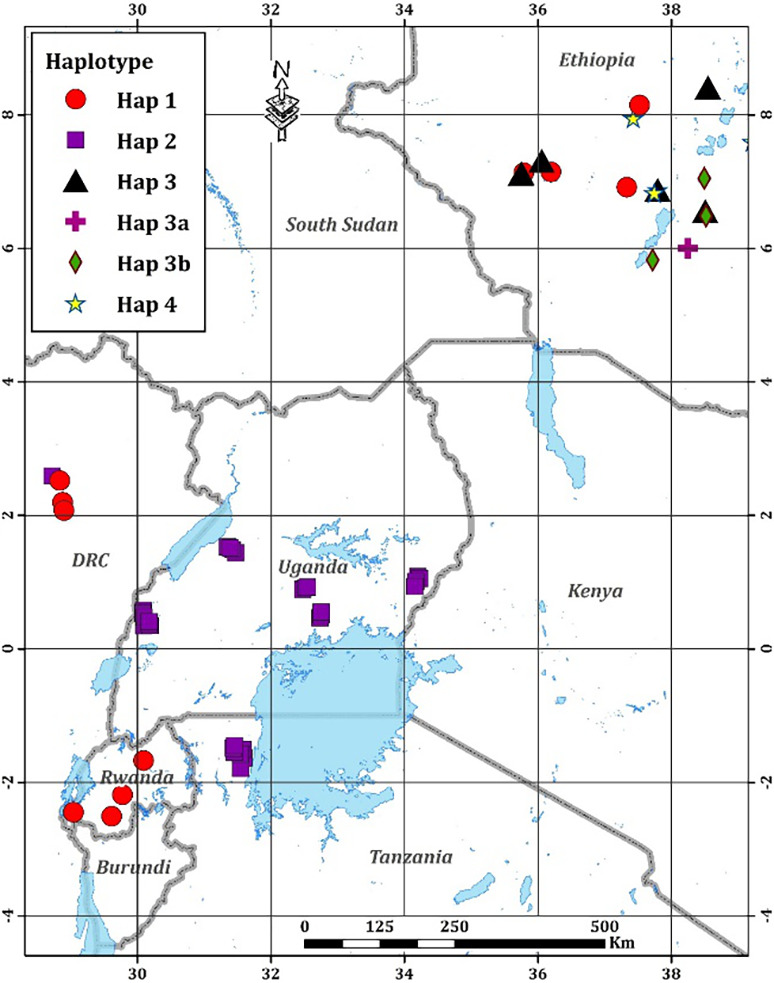
Geographical locations of *Xanthomonas vasicola* pv. *musacearum* isolates genotyped in this study. Red circle, isolates assigned to Haplotype 1 as defined in Table 1; purple squares, Haplotype 2; black triangles, Haplotype 3; purple cross, Haplotype 3a; green diamond, Haplotype 3b; yellow star, Haplotype 4

The most frequently observed haplotype was Haplotype 2, which included all the isolates from Uganda, Tanzania, and one from D. R. Congo (Figure 1). Haplotype 1 was observed in all isolates from Rwanda and some of the isolates from Ethiopia and D. R. Congo (Figure 1). Three previously unknown haplotypes, 3a, 3b, and 4, were discovered among Ethiopian isolates (Figure 1). Of the five haplotypes observed, Haplotype 1 was isolated from banana and enset, Haplotype 2 was only isolated from banana, Haplotype 3 was isolated only from enset, while Haplotype 4 was isolated from enset and maize (Table 3).

### 3.3 Genotyping by whole‐genome sequencing

**Figure 2:**
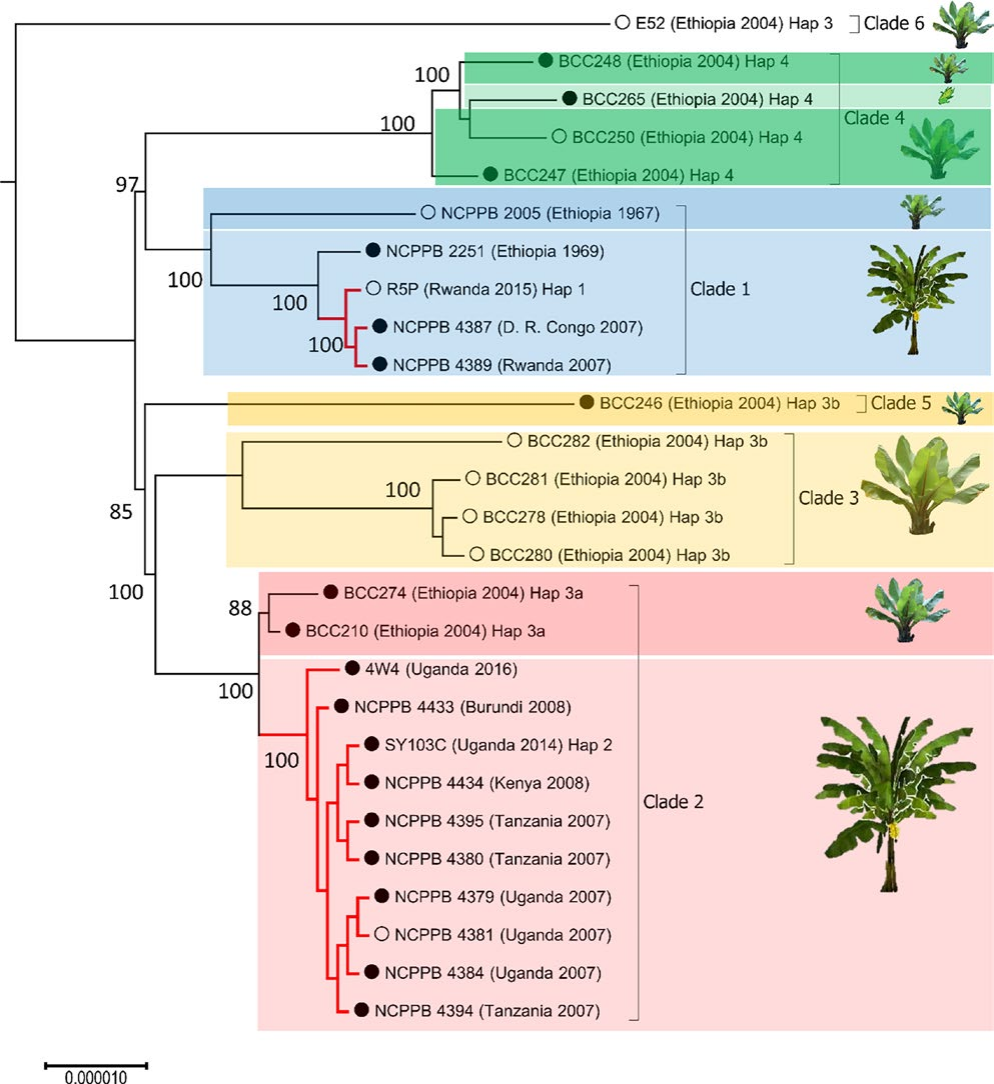
Genome‐based phylogenetic analysis of *Xanthomonas vasicola* pv. *musacearum* (Xvm) isolates. Phylogenetic tree analysis was performed on FastQ files using REALPHY (Bertels et al., 2014), applying the RaxML algorithm (Stamatakis, 2014) for tree constructing (NCPPB 4379 as reference genome). R5P, 4W4, SY103C, E52, and all BCC genomes were newly sequenced in the present study, while other Xvm genome sequences were published previously (Studholme et al., 2010; Wasukira et al., 2012). The tree was rooted on the genome sequence of the reference *X*. *vasicola* pv. *holcicola* NCPPB 2417 (not shown). The plasmid pXCM49 is present in the closed circle genomes, while absent from the open circle genomes. Red branches indicate genomes sampled out of Ethiopia. Node robustness is indicated by bootstrap values (percentages from 500 trials). All Ethiopian strains were isolated from enset, except NCPPB 2251 (banana) and BCC265 (maize). All other strains were isolated from banana

**Figure 3:**
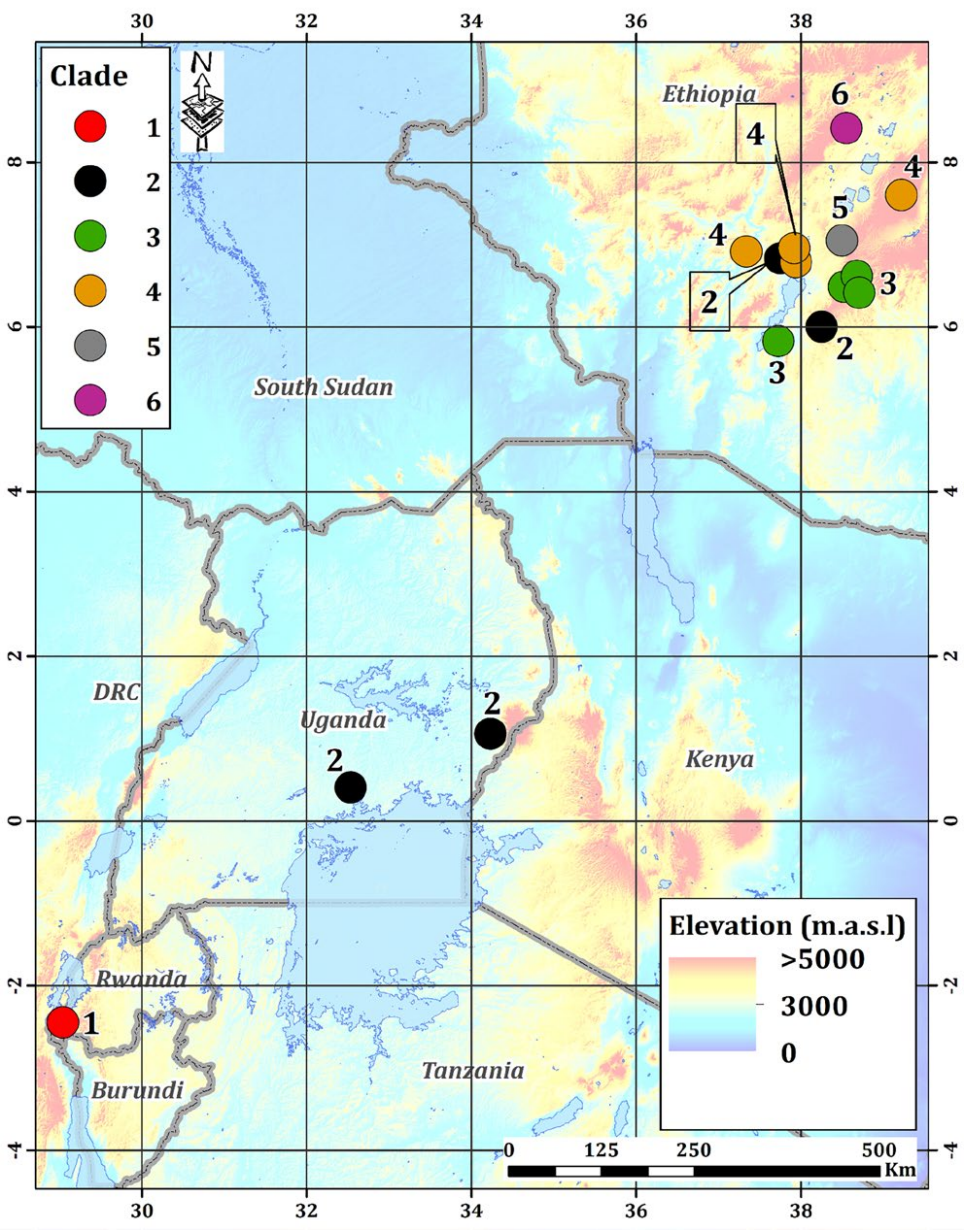
Geographical origin of the six clades in Ethiopia and eastern‐central Africa. Phylogenetic reconstruction from whole‐genome SNP data revealed six well‐defined genetic clades, with Clades 1 and 2 perfectly matching previously described sublineages SL 1 and SL 2 and four new clades (Clades 3–6). The Clade 1 Ethiopian isolates (NCPPB 2005, NCPPB 2251) had no GPS coordinates, and thus were not placed on the map

The results of the PCR‐RFLP assays revealed the existence of previously unknown Haplotypes 3 and 4. To position these unknown haplotypes within the Xvm phylogeny, we sequenced the genomes of 15 isolates, including representatives of each haplotype. We used REALPHY (Bertels et al., 2014) to perform phylogenetic analysis of these 15 genomes along with the 12 previously sequenced Xvm genomes (Wasukira et al., 2012) and a related *X*. *vasicola* pv. *vasculorum* genome as an outgroup. The resulting phylogenetic tree revealed six well‐defined genetic clusters that we called clades (Figure 2). A total of 1,170 SNPs, all within protein‐coding genes, differentiated the six clades (Table S2; Figure S1). Of these, 249 were silent, resulting in synonymous codon substitutions, and 617 were nonsilent, resulting in a change in the amino acid composition (Table S2). The most divergent clade was Clade 6, represented by a single genome (E52) sampled in 2015 in Ethiopian highlands within the Gurage district (woreda). Further branching separated Clades 1 and 4 on one side, and Clades 5, 3, and 2 in the other side (Figure 2). Clades 1 and 2 perfectly matched to the previously described sublineages SL 1 and SL 2, and were the only clades found outside Ethiopia. Clade 1 was thus found in Ethiopia, Rwanda, and D. R. Congo, whereas Clade 2 was found in Burundi, Uganda, Kenya, Tanzania, and Ethiopia. Interestingly, the Ethiopian Clade 2 genomes were grouped in an early diverging cluster within the clade. The correlation between SNP‐derived RFLP haplotypes and whole‐genome clades was contrasting. Haplotypes 1, 2, and 4 perfectly matched with Clades 1, 2, and 4, respectively (Table 2). The case of Haplotype 3 and its related 3a and 3b was different: Haplotype 3a correlated to Clade 2, whereas Haplotype 3b corresponded to Clades 3 and 5. Haplotype 3 corresponded to Clade 6 but is probably also composite. The geographical origins of the six clades reconstructed from the genome‐wide data of the 15 recently sequenced genomes are shown in Figure 3.

Only Clades 1 and 2 contained isolates from both enset and banana. Interestingly, the enset isolates in both clades (NCPPB 2005 in Clade 1, BCC210 and BCC274 in Clade 2) were significantly divergent (as judged by the bootstrap values) from the banana isolates.

All newly sequenced Xvm genomes were aligned to the finished genome assembly of strain NCPPB 4379 (GenBank: GCA_000277895.2) using Mauve v. 2.4.0. (Darling et al., 2004) (Figure S2). The alignment showed no evidence of any large‐scale genome rearrangements within the Xvm clade of *X*. *vasicola* (Figure S2).

### 3.4 Genome structure and gene content of Xvm clades

Analysis using Roary revealed that the Xvm pan‐genome comprises 4,467 gene clusters, of which 3,764 (84.26%) are core, that is, present in all analysed genome assemblies. A further 703 (15.74%) gene clusters were variable, that is, were absent from at least one genome assembly. Fifty‐six of these variable genes reside on the 49‐Mb plasmid pXCM49 (GenBank CP034656.1) found in most previously sequenced Xvm genomes but absent from several of the newly sequenced isolates (Figure 2); it is important to note that this plasmid carries no known virulence‐associated gene, such as adhesins and Type III effectors (nor Type II or Type IV effectors). Although Clade 3 genomes are all without the plasmid and Clade 2 genomes all carry the plasmid, the distribution of the plasmid pXCM49 across clades does not correlate entirely with phylogeny and might result from spontaneous curing under laboratory conditions. Gene content among strains ranged between 4,025 and 4,147 genes per genome.

There was no difference in the repertoires of Type III secretion effectors among sequenced Xvm; they each contained genes predicted to encode XopB, XopF1, XopG1, XopI, XopJ3, XopJ5, XopP, XopQ, XopV, XopZ1, XopAA, XopAB, XopAE, and XopAZ. Additionally, the draft genome assemblies encoded partial sequences for XopAD, but because of the difficulty in assembling this repetitive sequence de novo from relatively short reads, the status of this gene is ambiguous. The DNA sequences of most of the effector genes were identical across all six clades, with the exceptions of SNPs in *xopF1*, *xopK*, *xopAK*, *xopR*, *xopX*, *xopZ1*, *xopAE*, *xopAF2*, *xopAG*, and *xopP*. We found a short indel in *xopK*. These polymorphisms are illustrated in Figure S3a–m. There was no evidence of additional plasmids in any of these genomes. A full list of the 4,467 gene clusters, along with their presence/absence profiles across 18 genome assemblies and representative nucleotide sequences, is provided in Table S3. The pan‐genome is presented graphically in Figures S4 and S5.

## 4 DISCUSSION

Banana xanthomonas wilt is a relatively new epidemic, having emerged in East and Central Africa only in the 21st century. Understanding the origins and routes of transmission requires knowledge of genetic relationships between pathogen populations. Genetic variation in Xvm, the causative agent, is very limited (Odipio et al., 2009), thereby posing an obstacle to decipher population genetics of this organism. However, whole‐genome sequencing previously revealed several SNP loci that can distinguish several sublineages or clades. The reference study of Wasukira et al. (2012) described two sublineages within Xvm—one being hypothesized from Ethiopia (SL 1) and one of unknown origin (SL 2)—and proposed a list of diagnostic SNPs. We used some of these polymorphic loci to genotype a collection of isolates broadly spanning the geographical and temporal range of the epidemic. While our results are fully consistent with the two clades of Xvm on banana outside of Ethiopia being geographically separated as reported previously (Wasukira et al., 2012), this study updates the knowledge on Xvm diversity on several points.

First, we observed that both SL 1 and SL 2 clades are present in D. R. Congo, whereas previously the sole characterized isolate from that country belonged to SL 1. All isolates from Rwanda belonged to SL 1 whereas the isolates from Uganda and Tanzania belonged to SL 2. It appears that the geographical separation of SL 1 and SL 2 in Uganda, Tanzania, and Rwanda has persisted over time, because the isolates sampled from the current study were collected from a larger number of geographical locations in these countries about 10 years after those characterized by Wasukira et al. (2012).

Secondly, we demonstrated that the SL 2 clade also exists in Ethiopia, and the whole‐genome phylogeny indicated that Ethiopian SL 2/Clade 2 strains were basal to this cluster, suggesting that Clade 2 may also originate from Ethiopia.

Thirdly, we established that the level of Xvm phylogenetic diversity is much higher than previously known, because we described four new clades, all isolated from Ethiopia and from enset. Collectively, these findings further reinforce the hypothesis that Ethiopia is the centre of diversity of Xvm, and possibly its area of origin. Nevertheless, this pathovar remains apparently relatively monomorphic, with no more than a few hundred SNPs distinguishing any two isolates, significantly fewer than that observed in another pathovar of the same species (Aritua et al., 2007; Wasukira et al., 2014; Perez‐Quintero et al., 2020).

The six Xvm clades found in Ethiopia were isolated from enset cropped at highly variable altitudes, spanning from 1,089 to 2,670 m a.s.l. The question of the impact of environmental traits (rainfall and altitude) and cropping systems (diversity in Musaceae cultivars, overall plant diversity) on the divergence of Xvm remains an open question. Within this study, variables probably modulating the disease hotspots were not explored (Ocimati et al., 2019). According to Ocimati et al. (2019), altitude (i.e., temperature, and its effect on insect‐vectored spread) had a weak correlation to xanthomonas wilt attributed to the overriding impact of tool‐mediated spread that was part of the management covariate in their study. Ocimati et al. (2019) further identified Ethiopia and eastern D. R. Congo as areas that can currently be considered as xanthomonas wilt hotspots. This could explain in part the dispersion and diversity of the Xvm haplotypes.

The SNP‐derived RFLP typing system, despite not fully convergent with the genomic clades, has proven its usefulness in this study. This is definitely a simple, fast, and relatively cheap approach for identifying the different Xvm clades circulating within a country or a region. As previously reported (Nakato et al., 2019), the SNP typing system could be used in combination with the MLVA‐19 scheme within a hierarchical typing procedure, with the SNP markers being used to define the higher evolutionary groups at the clade level, and the MLVA‐19 scheme being used for outbreak investigations, regional surveillance, amount and directions of gene flows. The whole‐genome new clades were also correlated with the DAPC clusters identified using MLVA‐19, with Clade 1 grouping DAPC4, 5, and 11; Clade 2 grouping DAPC2, 3, and 8; and Clade 5 grouping DAPC 7 and 9. Clades 3, 4, and 6 corresponded to DAPC10 (Nakato et al., 2019, and data not shown).

It remains now to be determined whether these clades do differ in virulence towards different enset cultivars, and in host range towards enset, banana, and other Poaceae (specifically maize, which can be a natural host of Xvm, as observed in 2017 in Ethiopia). This study, and these genomic resources, pave the way for future studies addressing the evolutionary history of *X*. *vasicola* pv. *musacearum*, projects in functional genetics addressing the molecular basis of Xvm virulence on banana and enset, as well as breeding efforts for identifying efficient and durable banana and enset resistance sources.

## AUTHOR CONTRIBUTIONS

All authors planned and designed the research; G.V.N., E.M.W., and D.J.S. conducted the laboratory work; D.J.S., E.W., and G.V.N. analysed and interpreted the data; D.J.S., G.V.N., and E.W. wrote the manuscript, and G.B., M.G., T.A.C., E.M.W., and G.M. reviewed and refined the manuscript.

## Supporting information

Fig S1

Fig S2

Fig S3

Fig S4

Fig S5

Table S1

Table S2

Table S3

## Data Availability

Data that supports the findings of this study are available from the corresponding author on reasonable request.
