# Supplementary figures and images for: SNP‐based genotyping and whole‐genome sequencing reveal previously unknown genetic diversity in Xanthomonas vasicola pv. musacearum, causal agent of banana xanthomonas wilt, in its presumed Ethiopian origin
Source: Plant Pathol. 2020 Nov 27;70(3):534–43. doi: 10.1111/ppa.13308 (PMC7984043; doi:10.1111/ppa.13308)

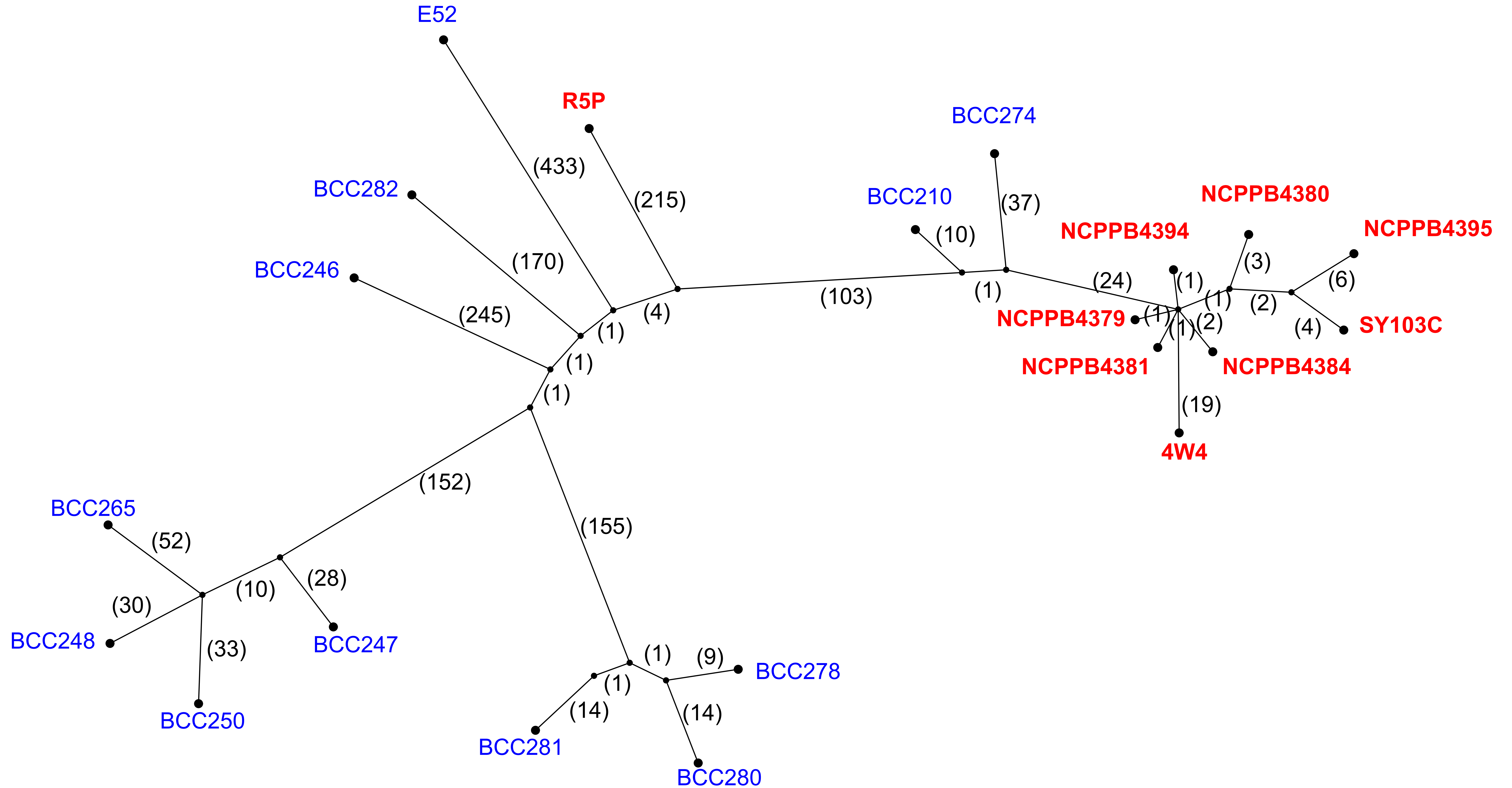

Supplement: Supplementary file 1 — Fig S1 [file PPA-70-534-s002.pdf]
