# Supplementary material for: SNP‐based genotyping and whole‐genome sequencing reveal previously unknown genetic diversity in Xanthomonas vasicola pv. musacearum, causal agent of banana xanthomonas wilt, in its presumed Ethiopian origin
Source: Plant Pathol. 2020 Nov 27;70(3):534–43. doi: 10.1111/ppa.13308 (PMC7984043; doi:10.1111/ppa.13308)

**Figure S2. Genome-wide alignment of the newly sequenced genomes of *Xanthomonas vasicola* pv. *musacearum*.** We re-ordered that scaffolds of the draft assemblies against the finished genome assembly of strain NCPPB 4379 (GenBank: GCA_000277895.2) using Mauve version 2.4.0. Long vertical lines represent contig limits.


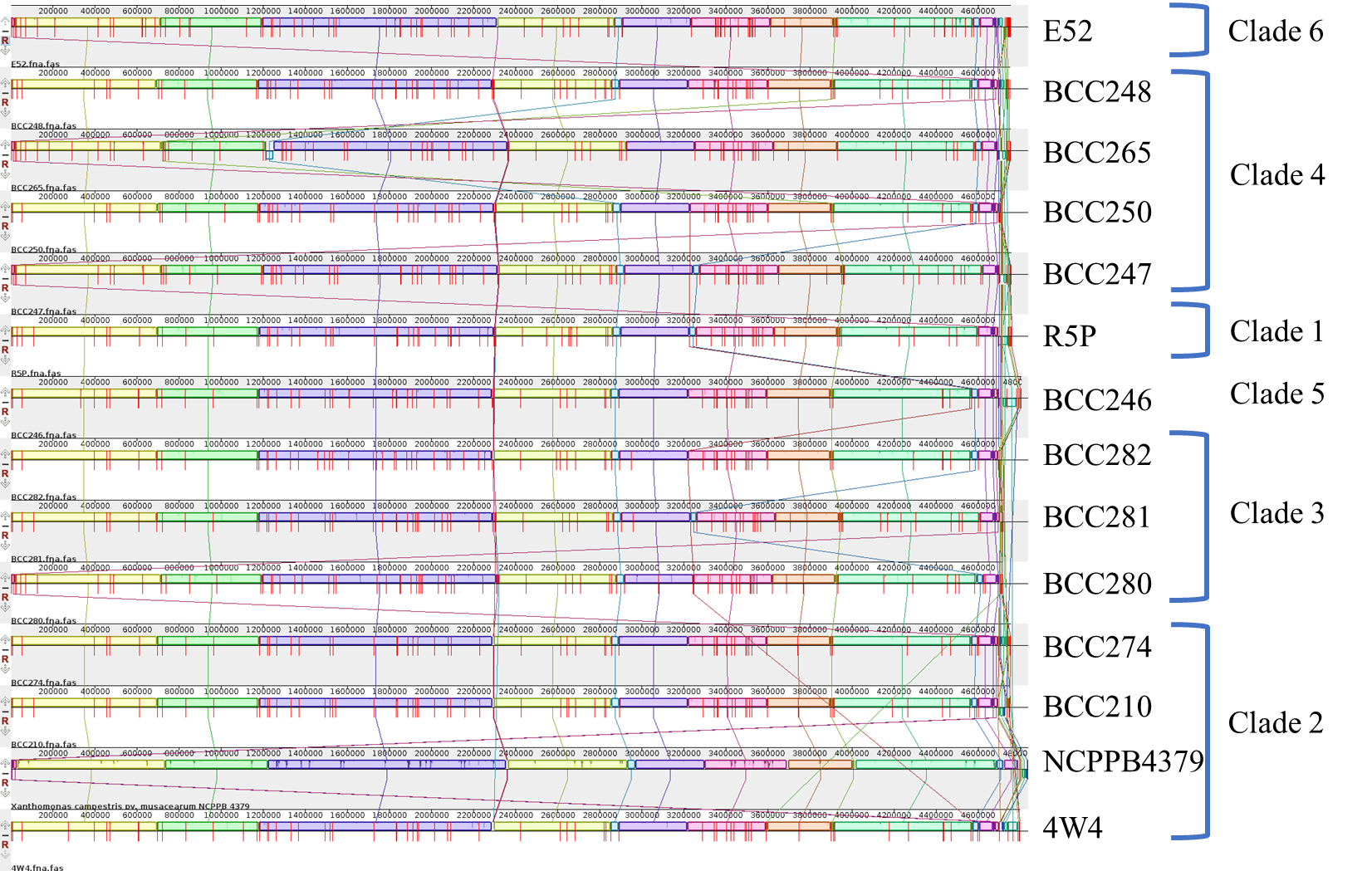

Supplement: Supplementary file 2 — Fig S2 [file PPA-70-534-s007.docx]
