# Supplementary material for: SNP‐based genotyping and whole‐genome sequencing reveal previously unknown genetic diversity in Xanthomonas vasicola pv. musacearum, causal agent of banana xanthomonas wilt, in its presumed Ethiopian origin
Source: Plant Pathol. 2020 Nov 27;70(3):534–43. doi: 10.1111/ppa.13308 (PMC7984043; doi:10.1111/ppa.13308)
Supplement: Supplementary file 3 — Fig S3 [file PPA-70-534-s006.pptx]

## Slide 1
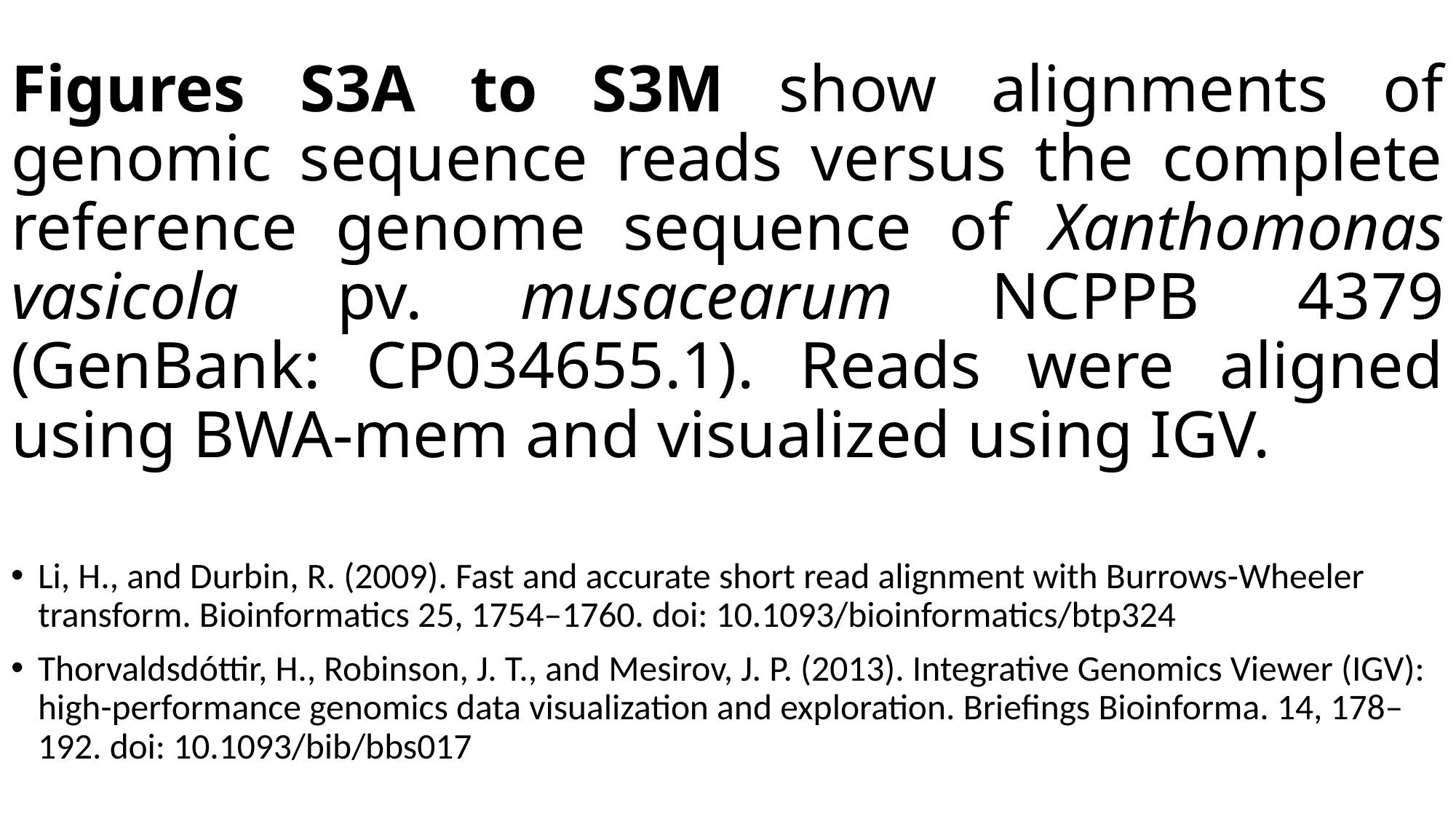

# Figures S3A to S3M show alignments of genomic sequence reads versus the complete reference genome sequence of Xanthomonas vasicola pv. musacearum NCPPB 4379 (GenBank: CP034655.1). Reads were aligned using BWA-mem and visualized using IGV.
﻿﻿Li, H., and Durbin, R. (2009). Fast and accurate short read alignment with Burrows-Wheeler transform. Bioinformatics 25, 1754–1760. doi: 10.1093/bioinformatics/btp324
﻿Thorvaldsdóttir, H., Robinson, J. T., and Mesirov, J. P. (2013). Integrative Genomics Viewer (IGV): high-performance genomics data visualization and exploration. Briefings Bioinforma. 14, 178–192. doi: 10.1093/bib/bbs017

## Slide 2
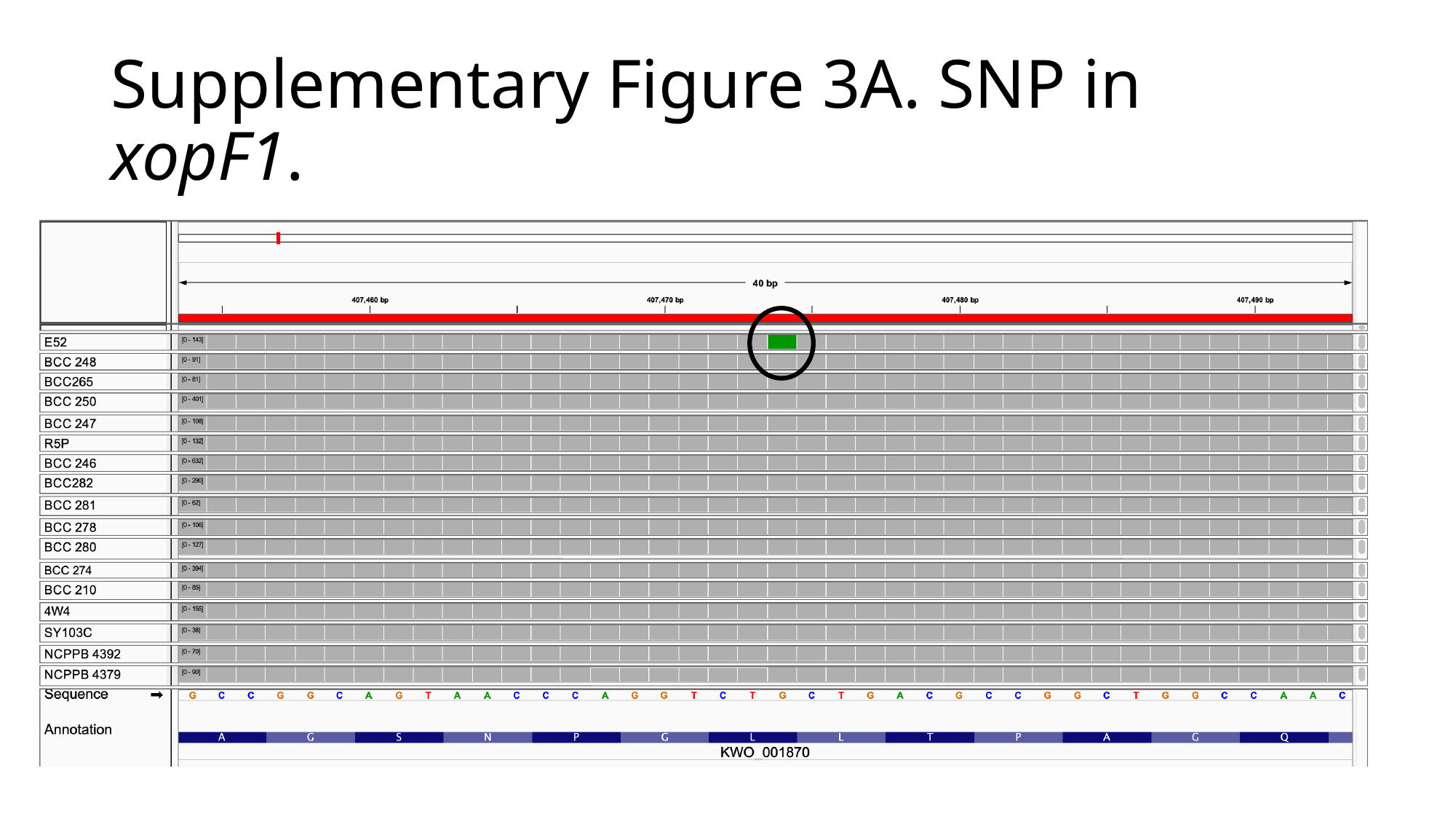

# Supplementary Figure 3A. SNP in xopF1.

## Slide 3
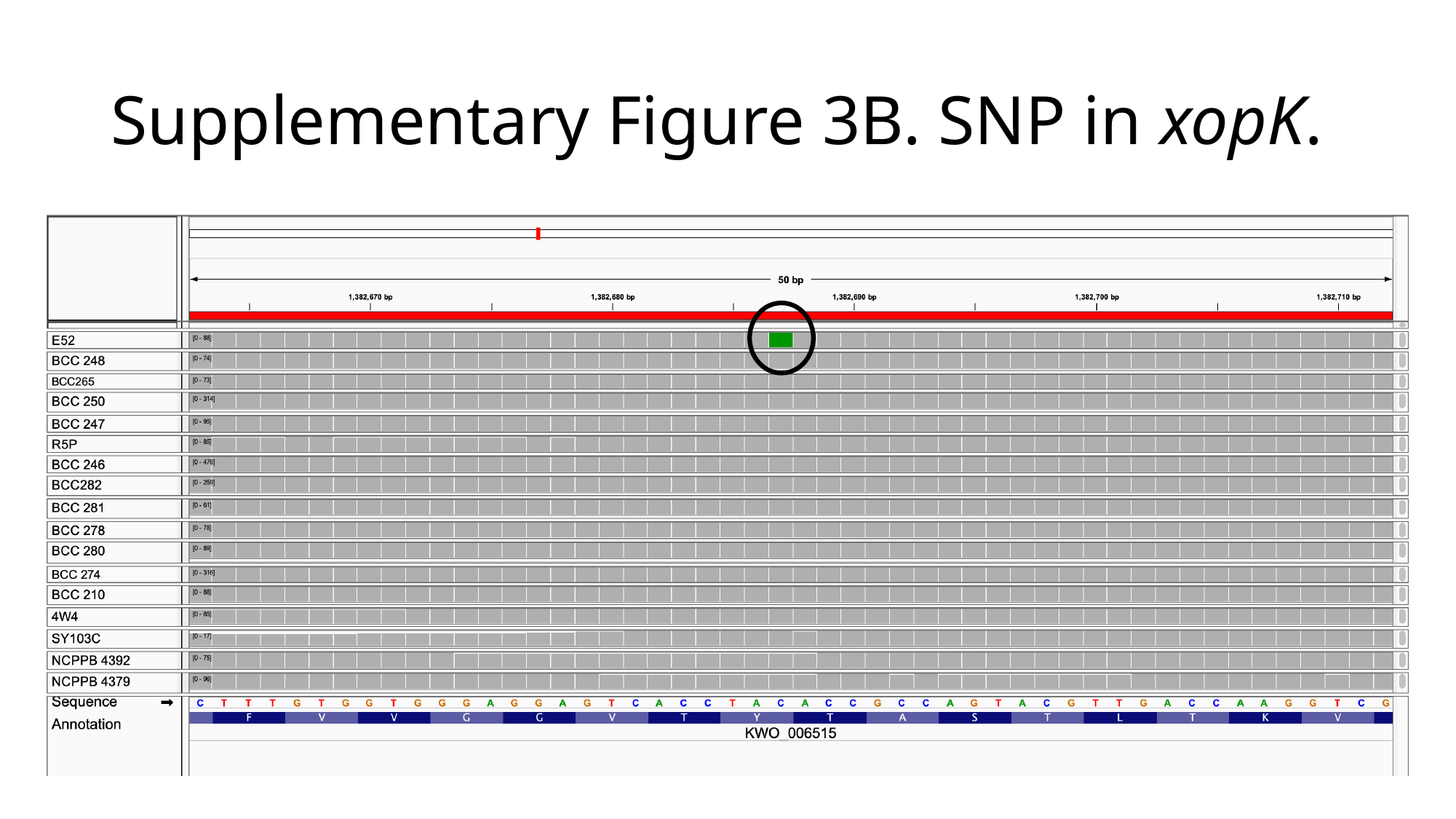

# Supplementary Figure 3B. SNP in xopK.

## Slide 4
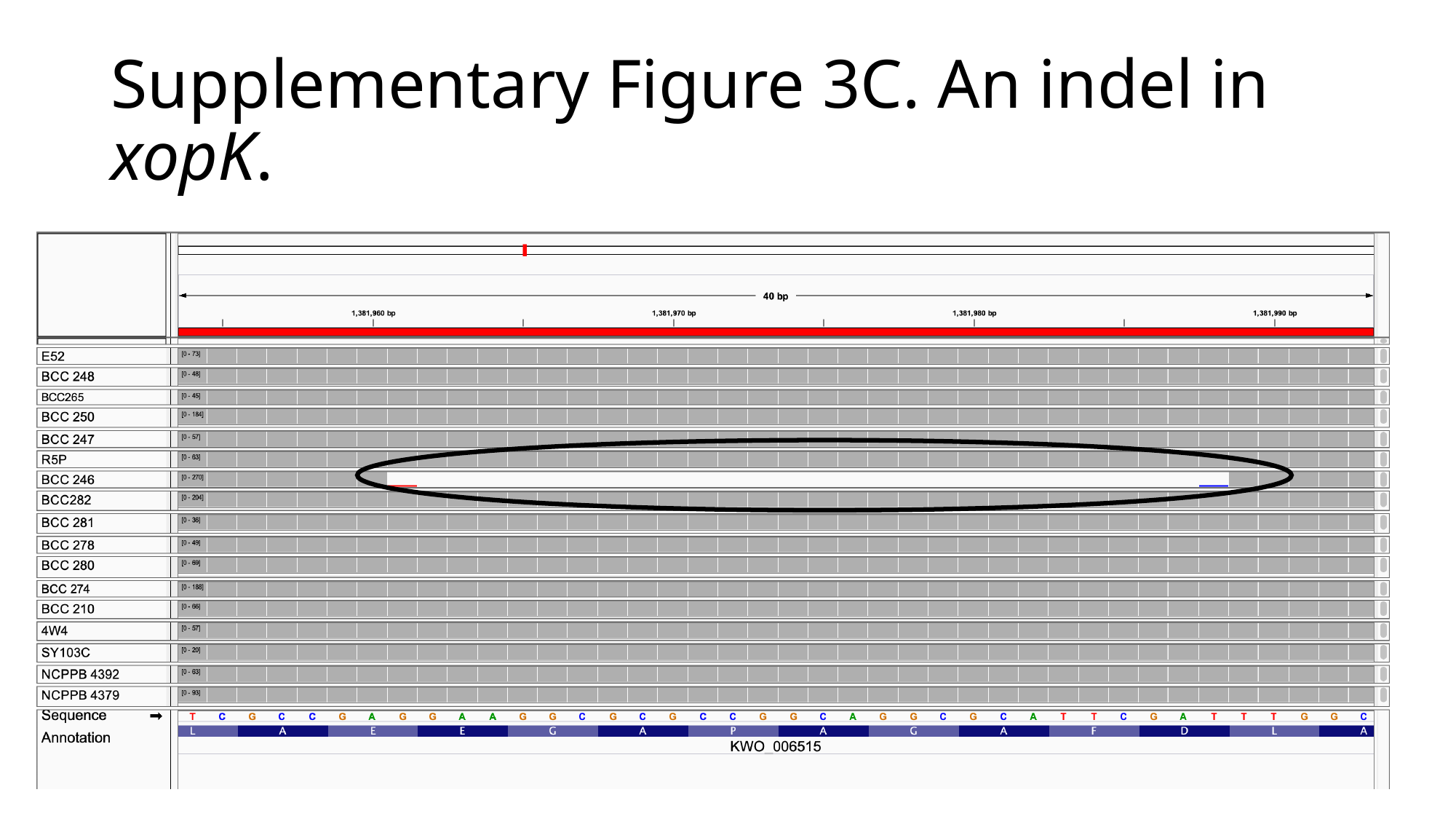

# Supplementary Figure 3C. An indel in xopK.

## Slide 5
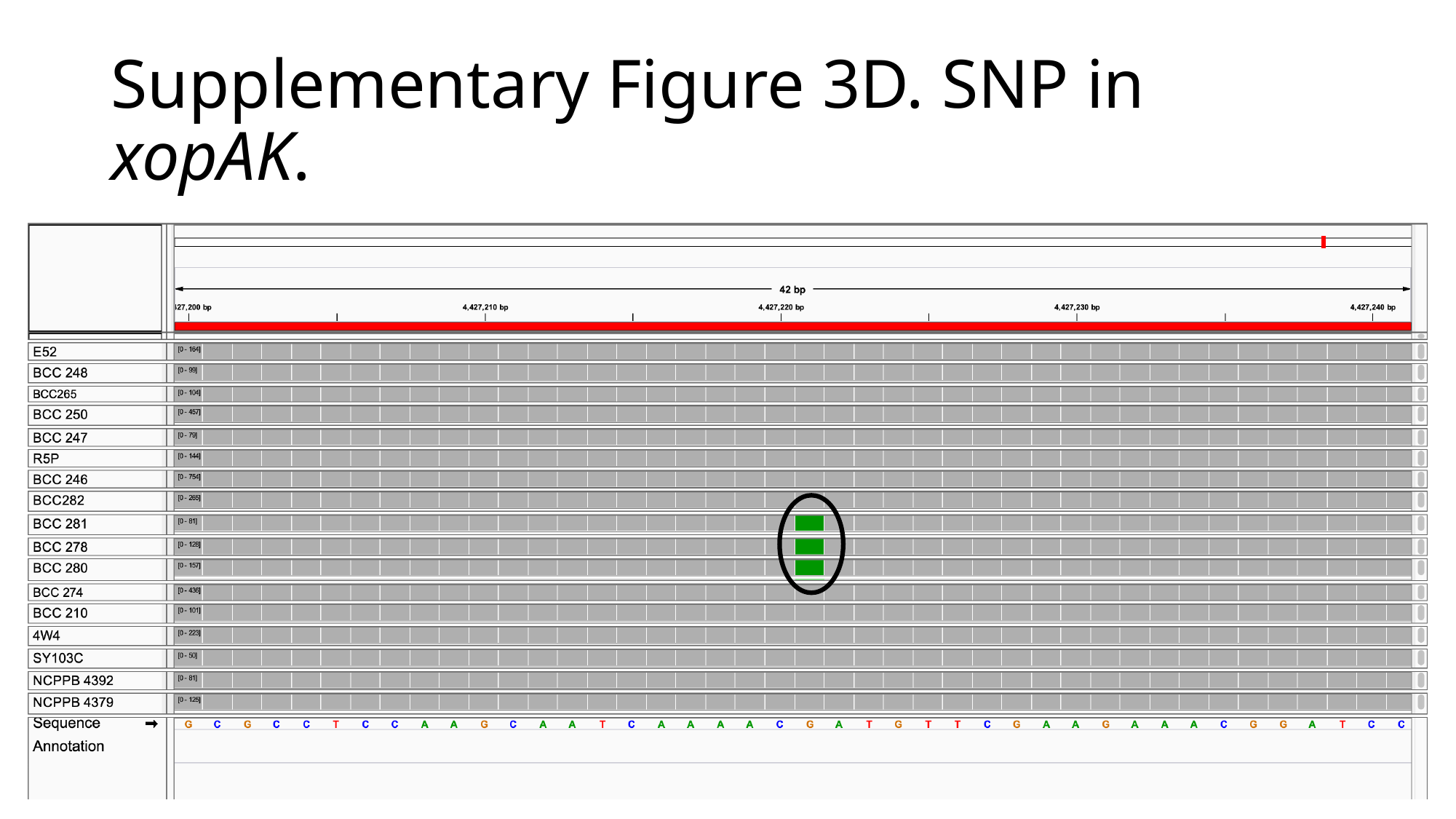

# Supplementary Figure 3D. SNP in xopAK.

## Slide 6
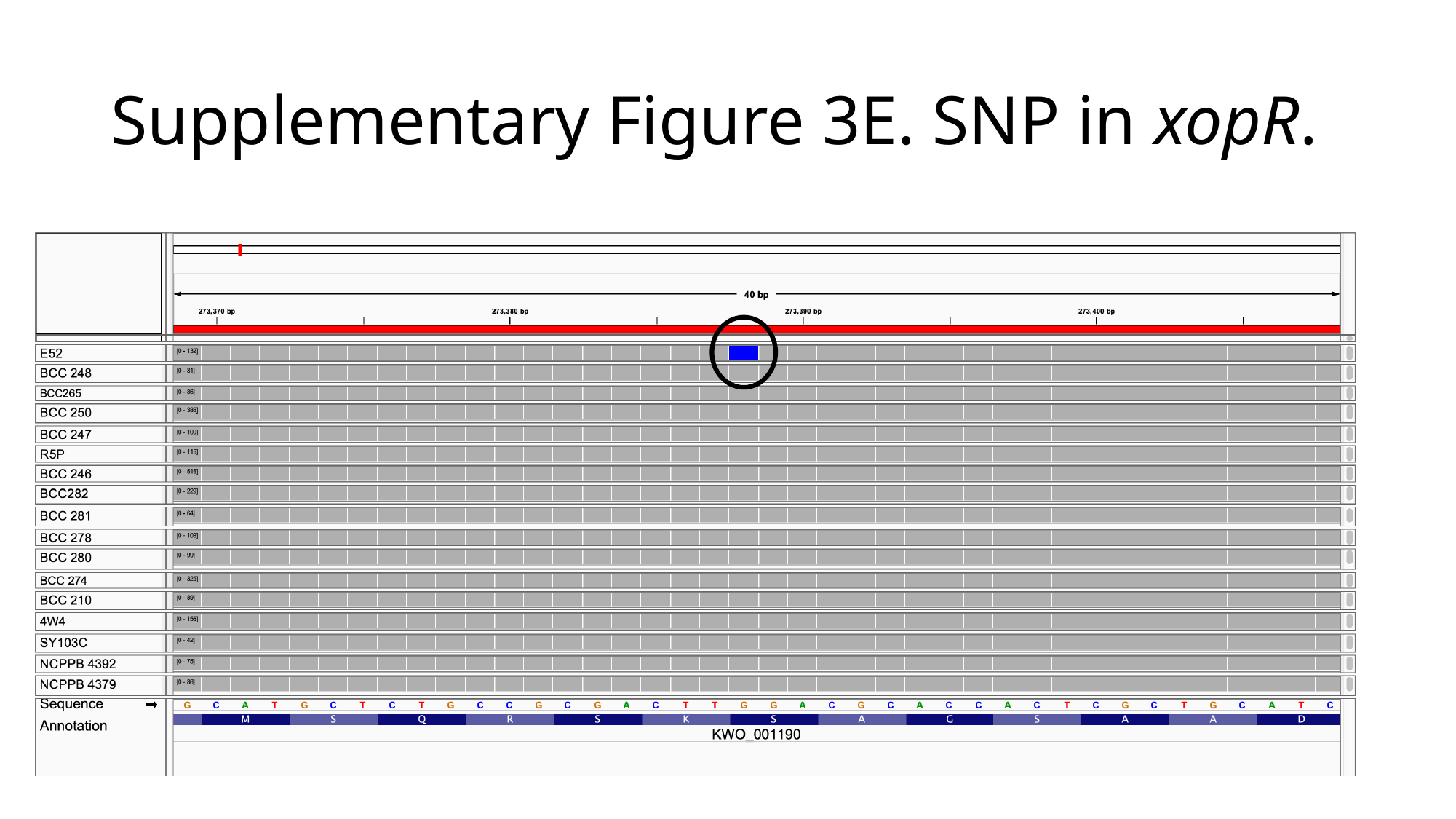

# Supplementary Figure 3E. SNP in xopR.

## Slide 7
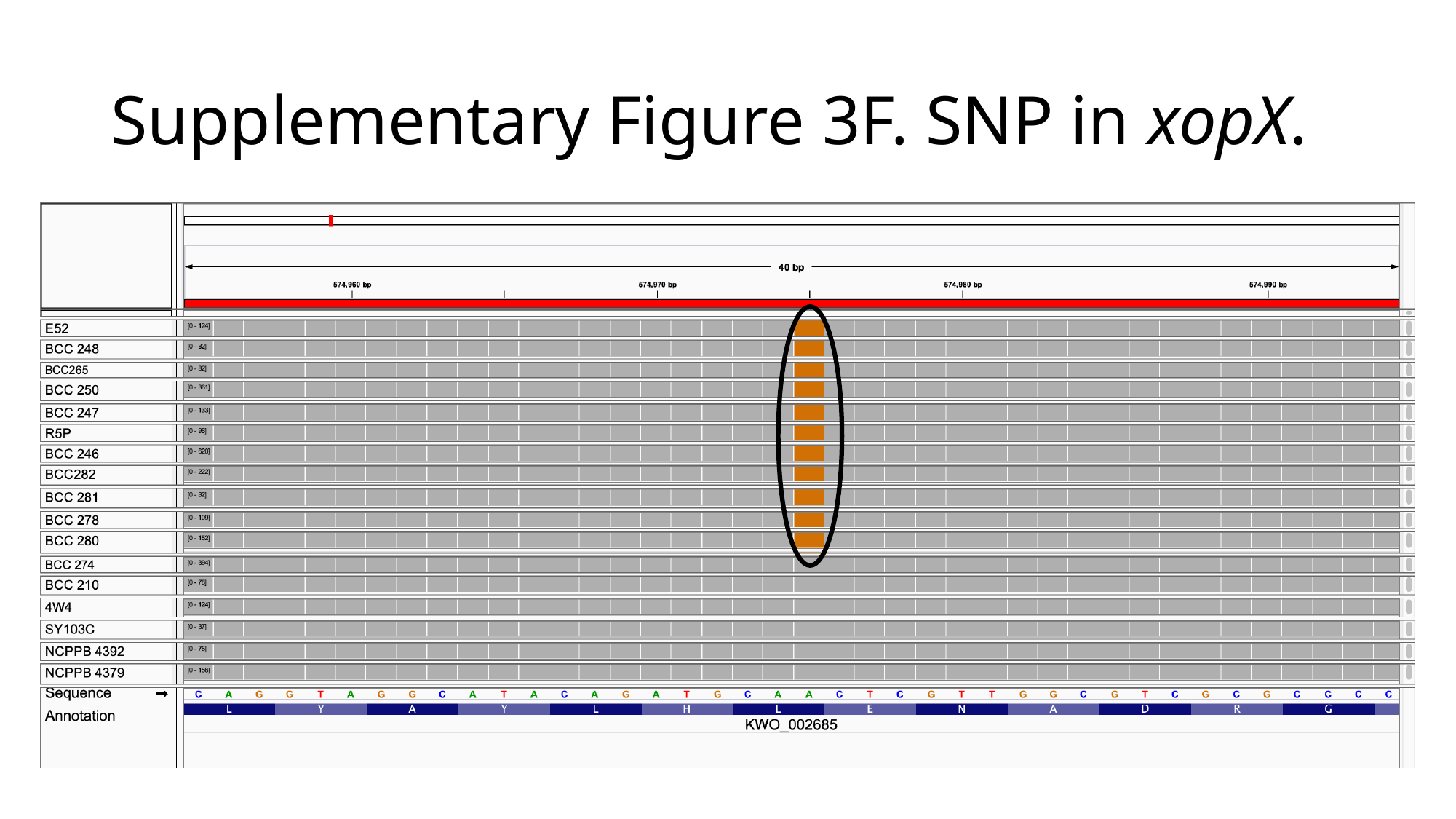

# Supplementary Figure 3F. SNP in xopX.

## Slide 8
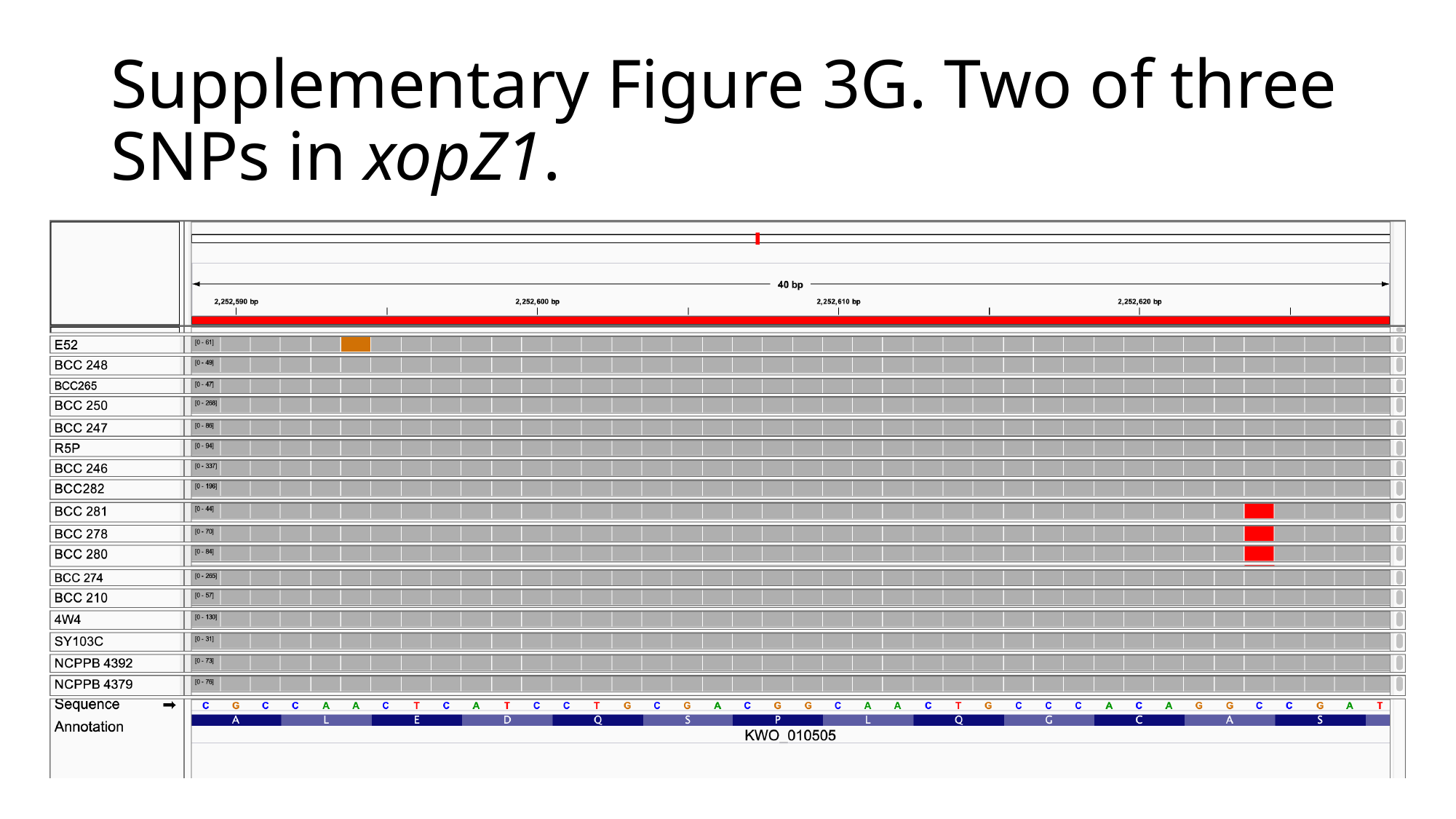

# Supplementary Figure 3G. Two of three SNPs in xopZ1.

## Slide 9
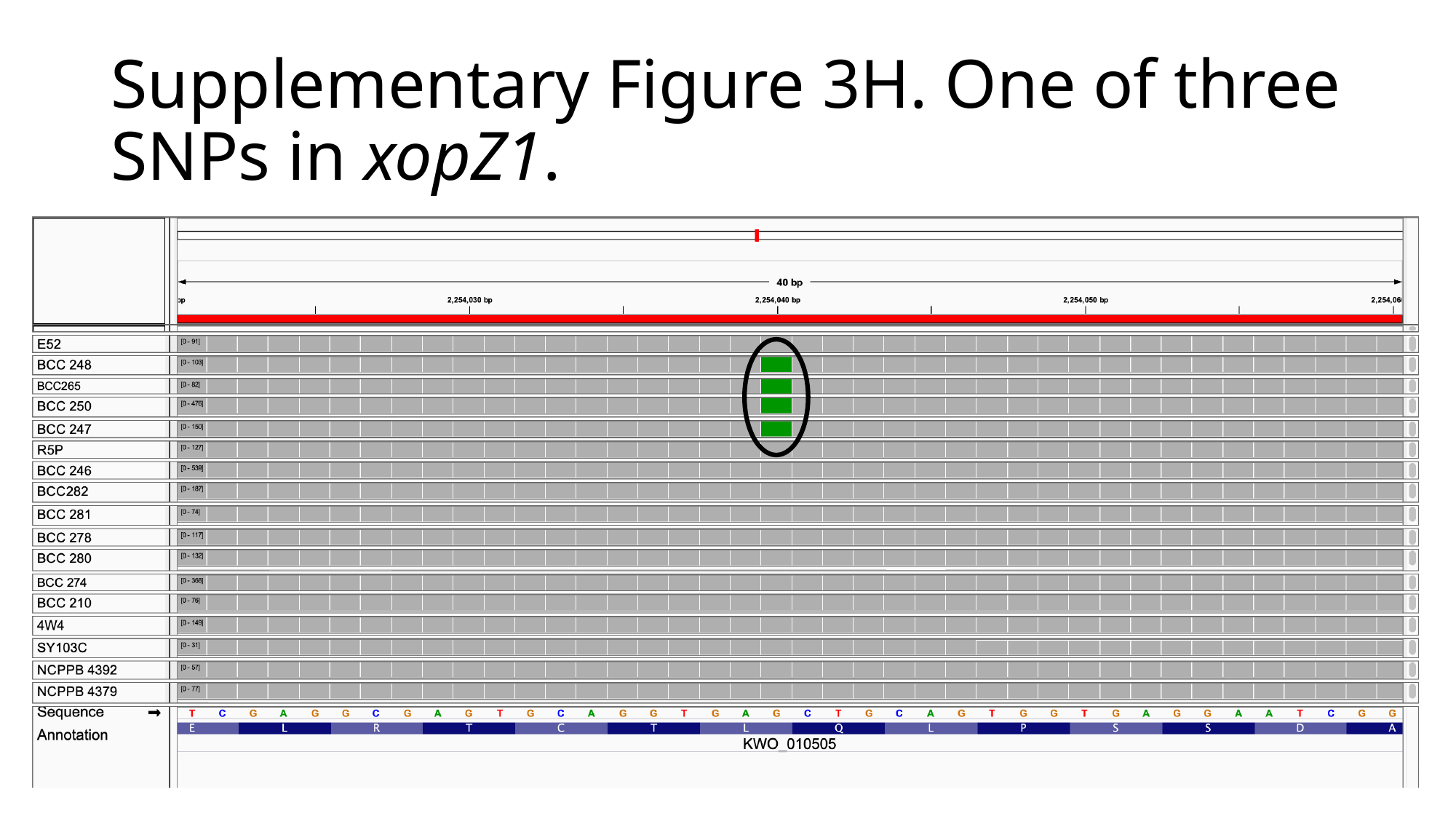

# Supplementary Figure 3H. One of three SNPs in xopZ1.

## Slide 10
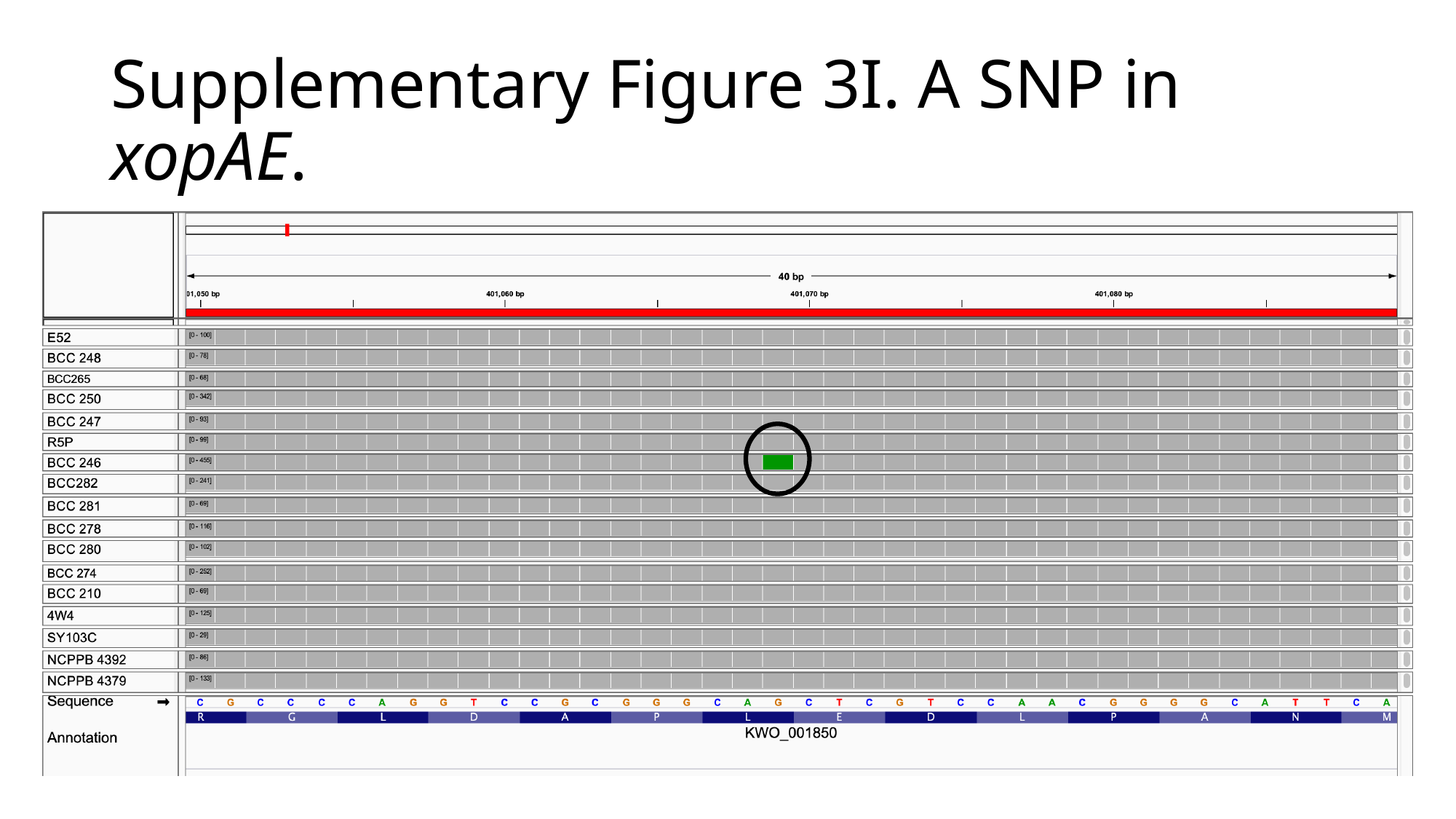

# Supplementary Figure 3I. A SNP in xopAE.

## Slide 11
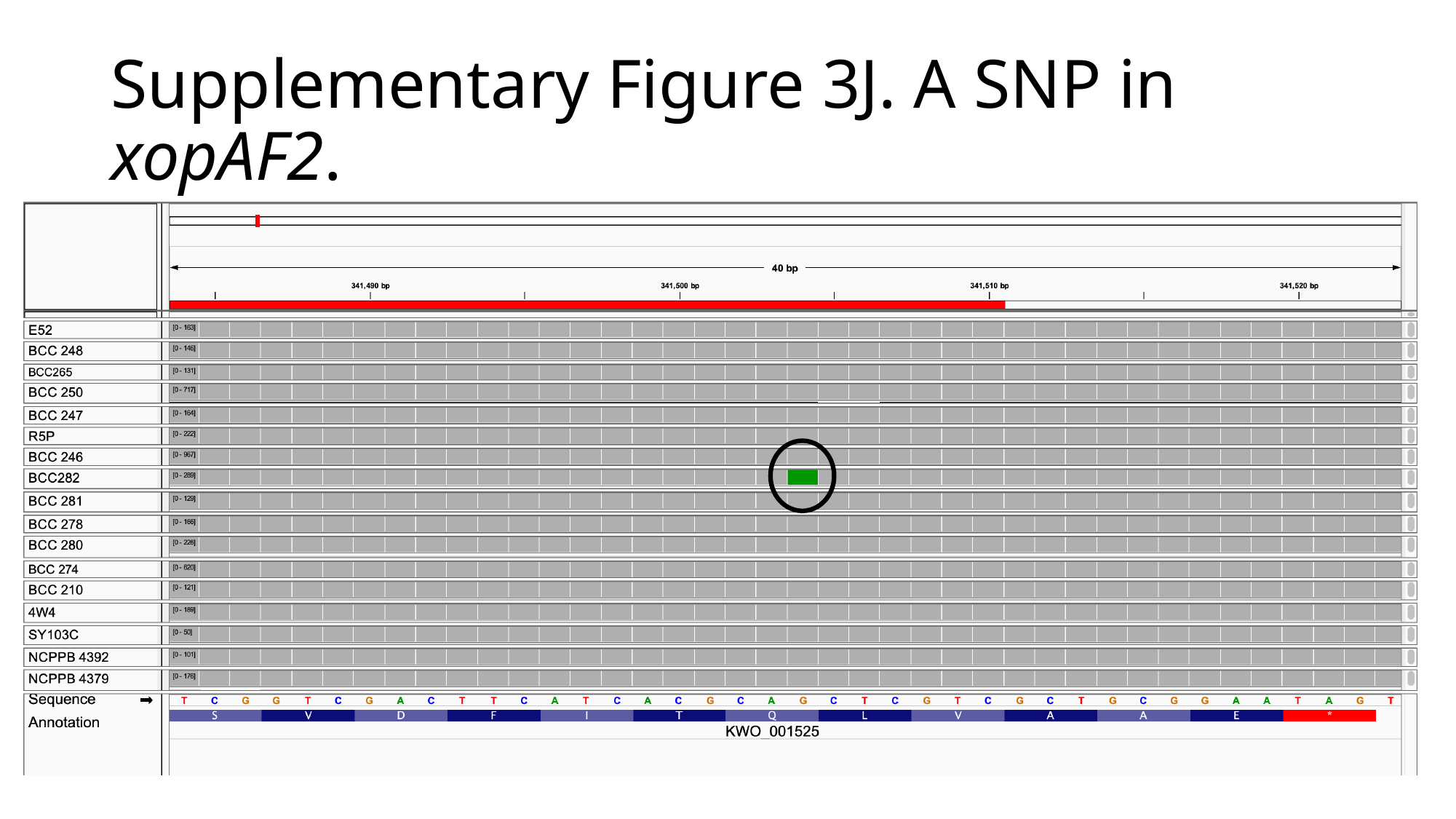

# Supplementary Figure 3J. A SNP in xopAF2.

## Slide 12
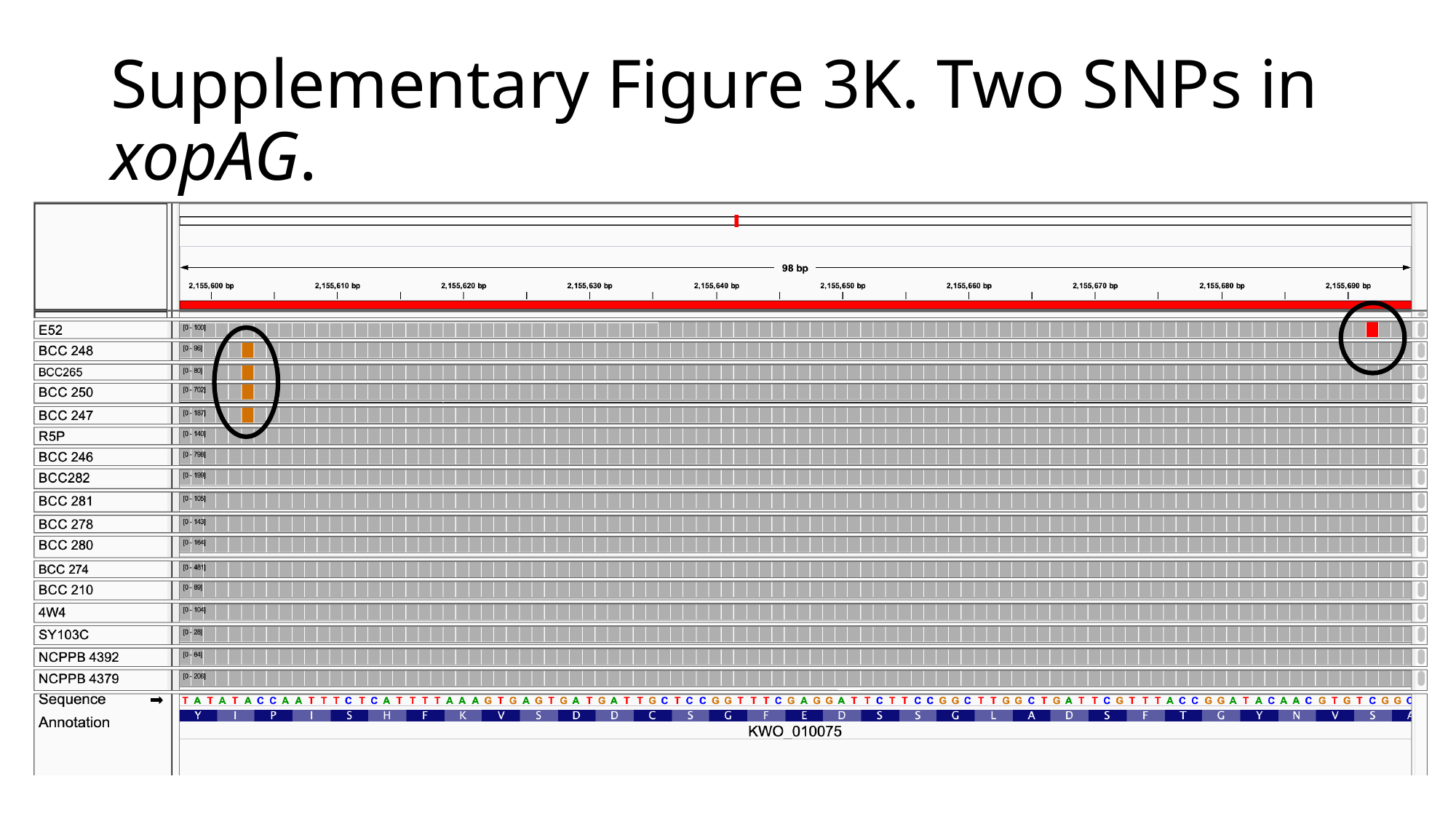

# Supplementary Figure 3K. Two SNPs in xopAG.

## Slide 13
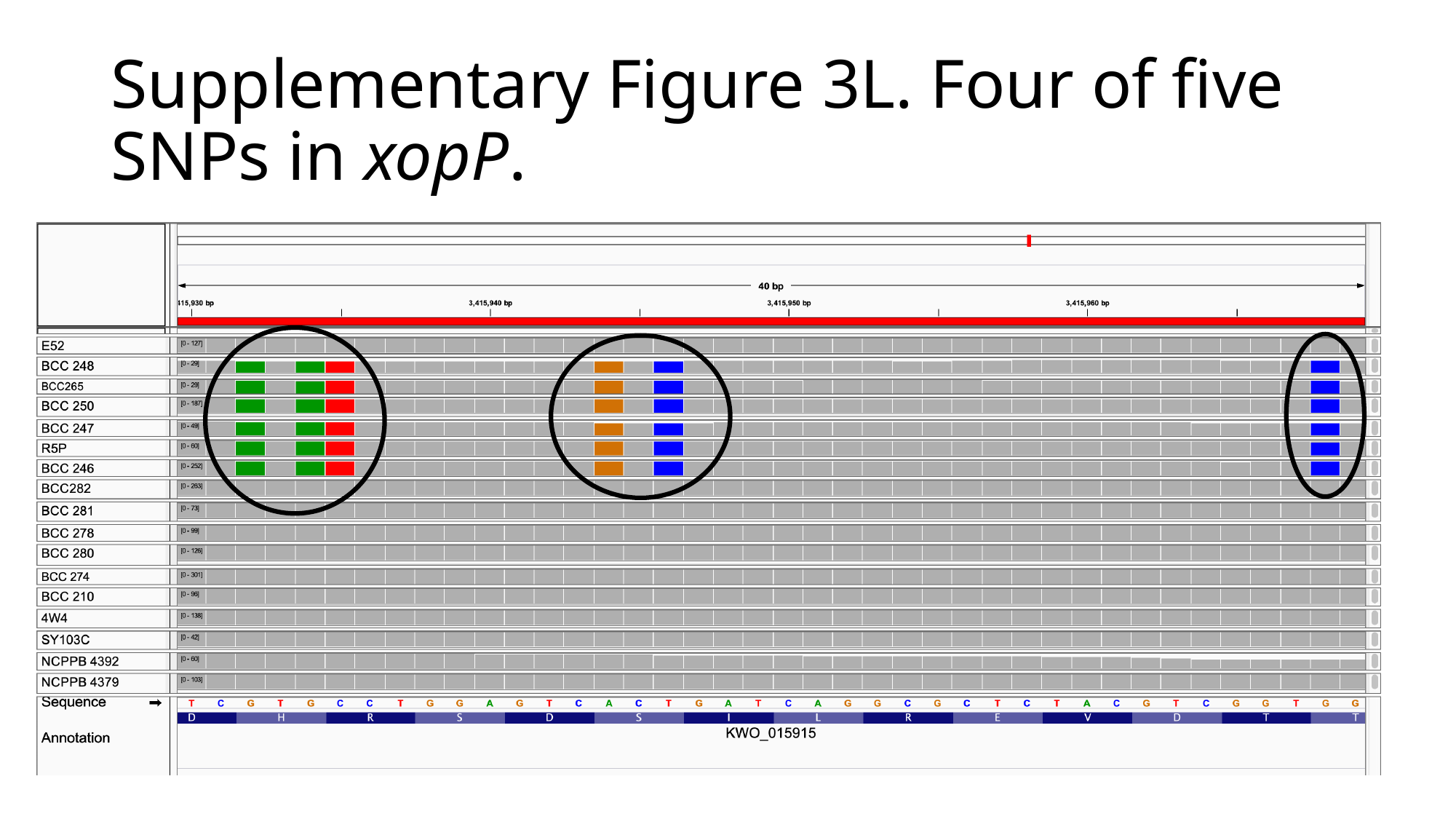

# Supplementary Figure 3L. Four of five SNPs in xopP.

## Slide 14
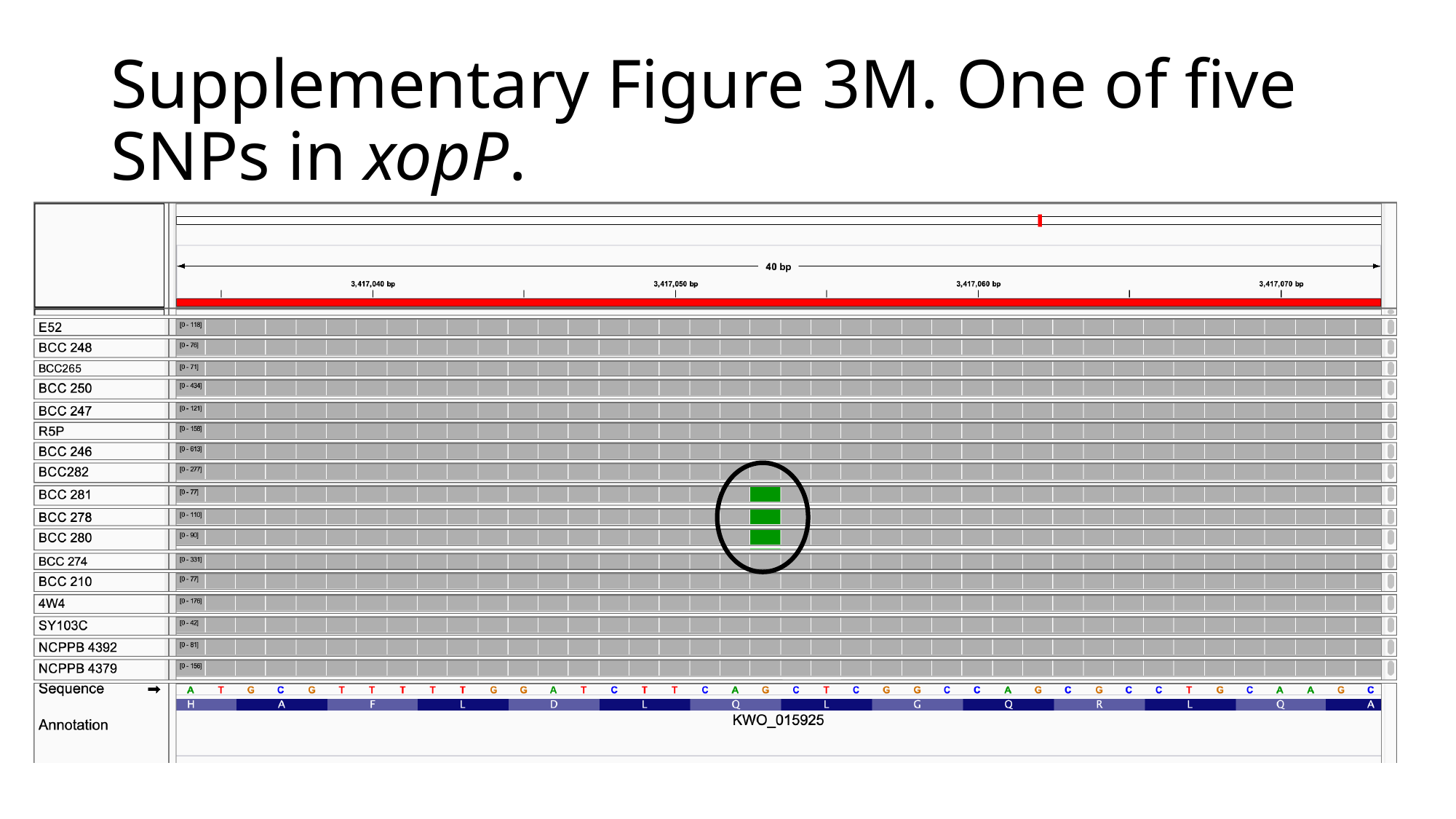

# Supplementary Figure 3M. One of five SNPs in xopP.
