# Supplementary material for: SNP‐based genotyping and whole‐genome sequencing reveal previously unknown genetic diversity in Xanthomonas vasicola pv. musacearum, causal agent of banana xanthomonas wilt, in its presumed Ethiopian origin
Source: Plant Pathol. 2020 Nov 27;70(3):534–43. doi: 10.1111/ppa.13308 (PMC7984043; doi:10.1111/ppa.13308)

**Figure S4. Heatmap of the pan-genome of *Xanthomonas vasicola* pv. *musacearum*.** The heatmap shows the distribution of the genes within the different *Xanthomonas vasicola* pv. *musacearum* genomes. The pan-genome was calculated using Roary version 3.13.0 (Page *et al*., 2015) after annotating the genome assemblies with Prokka version 1.14.5 (Seemann, 2014). The heatmap was generated using the roary_plots.py script by Marco Galardini (<https://github.com/sanger-pathogens/Roary/tree/master/contrib/roary_plots>).


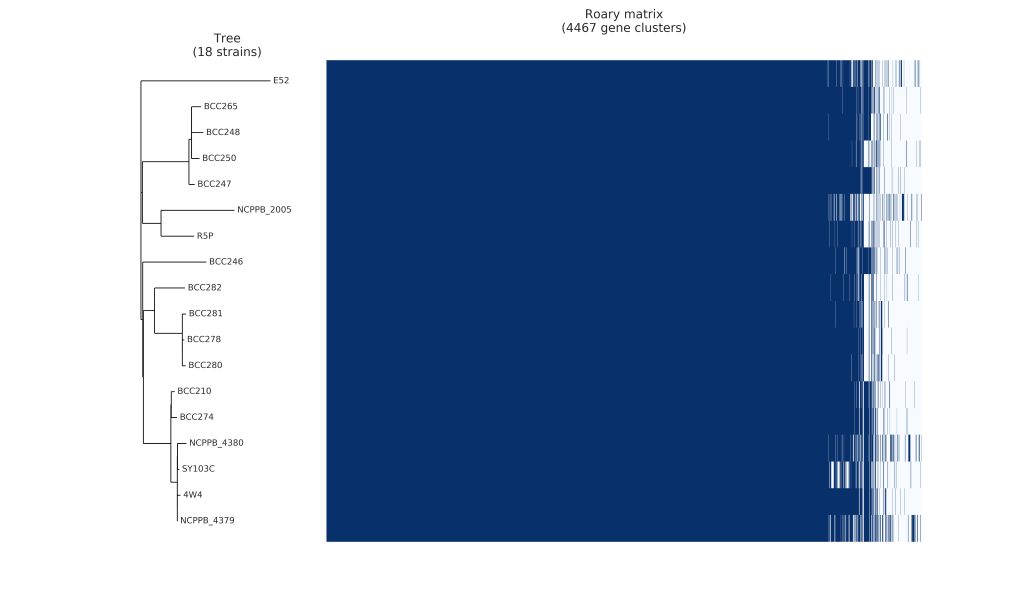

Supplement: Supplementary file 4 — Fig S4 [file PPA-70-534-s005.docx]
