# Supplementary material for: SNP‐based genotyping and whole‐genome sequencing reveal previously unknown genetic diversity in Xanthomonas vasicola pv. musacearum, causal agent of banana xanthomonas wilt, in its presumed Ethiopian origin
Source: Plant Pathol. 2020 Nov 27;70(3):534–43. doi: 10.1111/ppa.13308 (PMC7984043; doi:10.1111/ppa.13308)

**Figure S5. Piechart of the pan-genome of *Xanthomonas vasicola* pv. *musacearum***. The pie-chart shows the numbers of genes broken down into the core, soft core, shell and cloud. The pan-genome was calculated using Roary version 3.13.0 (Page *et al*., 2015) after annotating the genome assemblies with Prokka version 1.14.5 (Seemann, 2014). The pie-chart was generated using the roary_plots.py script by Marco Galardini (<https://github.com/sanger-pathogens/Roary/tree/master/contrib/roary_plots>).


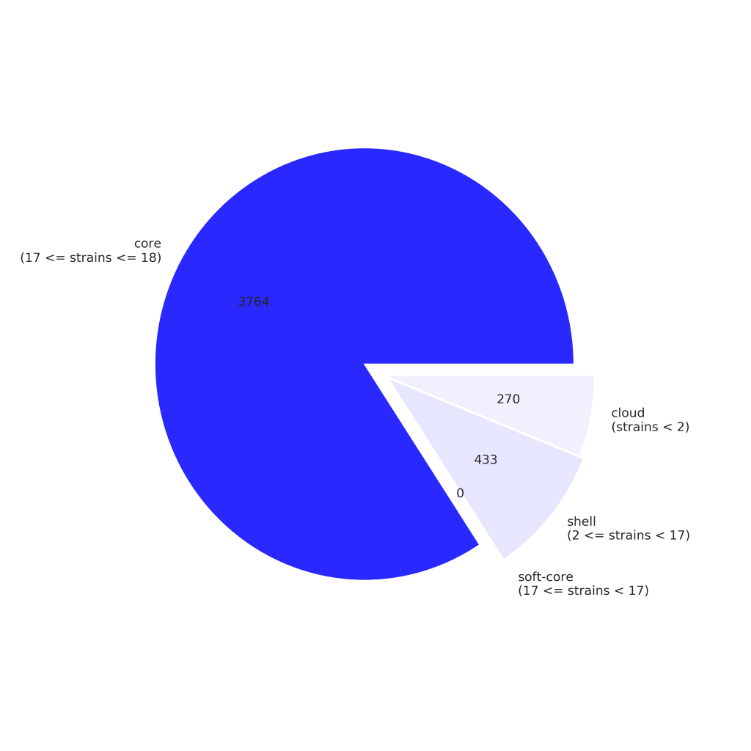

Supplement: Supplementary file 5 — Fig S5 [file PPA-70-534-s004.docx]
